# The Systematics, Reproductive Biology, Biochemistry, and Breeding of Sea Buckthorn—A Review

**DOI:** 10.3390/genes14122120

**Published:** 2023-11-24

**Authors:** Hilde Nybom, Chengjiang Ruan, Kimmo Rumpunen

**Affiliations:** 1 Department of Plant Breeding–Balsgård, Swedish University of Agricultural Sciences, 29194 Kristianstad, Sweden; 2 Key Laboratory of Biotechnology and Bioresources Utilization, Ministry of Education, Institute of Plant Resources, Dalian Minzu University, Dalian 116600, China; ruan@dlnu.edu.cn; 3 Department of Plant Breeding, Swedish University of Agricultural Sciences, 23053 Alnarp, Sweden; kimmo.rumpunen@slu.se

**Keywords:** *Hippophae*, chemical contents, cultivar development, DNA markers, genetics, medicinal plant, systematics

## Abstract

Both the fruit flesh and seeds of sea buckthorn have multiple uses for medicinal and culinary purposes, including the valuable market for supplementary health foods. Bioactive compounds, such as essential amino acids, vitamins B, C, and E, carotenoids, polyphenols, ursolic acid, unsaturated fatty acids, and other active substances, are now being analyzed in detail for their medicinal properties. Domestication with commercial orchards and processing plants is undertaken in many countries, but there is a large need for improved plant material with high yield, tolerance to environmental stress, diseases, and pests, suitability for efficient harvesting methods, and high contents of compounds that have medicinal and/or culinary values. Applied breeding is based mainly on directed crosses between different subspecies of *Hippophae rhamnoides*. DNA markers have been applied to analyses of systematics and population genetics as well as for the discrimination of cultivars, but very few DNA markers have as yet been developed for use in selection and breeding. Several key genes in important metabolic pathways have, however, been identified, and four genomes have recently been sequenced.

## 1. Introduction

**Figure 1:**
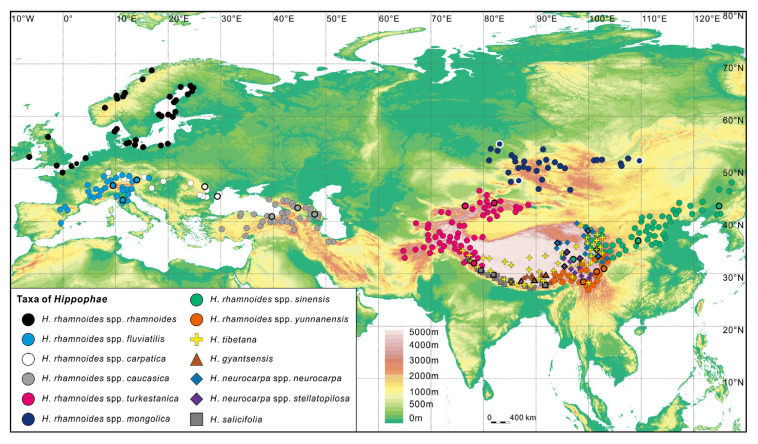
Geographic distribution of *Hippophae* L. (reprinted with permission from [1]), 2018, Jia and Bartish.

The genus *Hippophae* L. belongs to the small plant family Elaeagnaceae. The deciduous shrubs or small trees, usually 2–5 m high (occasionally over 10 m), are wind-pollinated and dioecious (male and female flowers are produced on separate plants). The yellow to orange or sometimes reddish to red fruits contain one seed each, and these seeds are often dispersed by birds and other frugivorous animals. The most well-known species, *Hippophae rhamnoides*, commonly known as sea buckthorn, is widely distributed over China, the Indian Himalayas, Central Asia, and Russia, as well as large parts of Europe (Figure 1).

Both the fruit flesh and the seeds of sea buckthorn have multiple uses for medicinal and culinary purposes, including the valuable market for supplementary health foods [2]. The sea buckthorn industry has been thriving in Russia ever since the bioactive compounds started to become appreciated. By contrast, industrial use is somewhat more recent in China, despite several centuries of traditional use in Chinese medicine. Domestication with commercial orchards and/or processing plants is now undertaken for both culinary and medicinal products in, e.g., Mongolia, Nepal, Tajikistan, India, Iran, Belarus, Ukraine, Turkey, Greece, Romania, Germany, Finland, Sweden, and the Baltic countries, as well as in some other parts of Europe. Sea buckthorn has also been introduced to other countries like Canada, the USA, Bolivia, Chile, South Korea, and Japan.

The sea buckthorn is a typical pioneer plant and prefers open habitats with sandy or rocky free-draining soils, high insolation, and restricted competition from other species. Plants are spread efficiently via seeds and root suckers. Sea buckthorn plants have root nodules that contain nitrogen-fixating bacteria (the actinomycete *Frankia*), allowing them to thrive in nutritionally deficient soils. Due to its high tolerance to extreme conditions (both very low and very high temperatures, drought, salinity, and poor soils), sea buckthorn is often grown as protection against wind, for prevention of sand drift, for conservation of soil and water, and to adjust microclimate conditions, especially in China but also in India, Russia, Canada, and Bolivia [3].

In December 2020, the total sea buckthorn acreage, including both wild and cultivated plants, was estimated to amount to approximately 2.33 million ha in the world (http://www.isahome.net/news.php?id=586, accessed on 12 September 2023). Of these, about 2.07 million ha were found in China (0.77 million ha wild and 1.35 million ha planted), 16,300 ha in India, 15,000 ha in Romania, 20,000 ha in Mongolia, ~6000 ha in Russia, and 5700 ha in Pakistan.

The increasing interest in sea buckthorn is mainly centered around its properties as a medicinal and culinary plant. In addition to their long-time use in traditional Chinese medicine for, e.g., calming coughs, aiding digestion, improving blood circulation and alleviating pain, recent pharmacological studies have shown that crude extracts or compounds have anti-inflammatory, antioxidant, hepatoprotective, anticancer, hypoglycemic, hypolipidemic, neuroprotective, and antibacterial properties (review in [3,4]).

Hundreds of bioactive compounds, such as essential amino acids, vitamins B, C, and E, carotenoids, polyphenols, ursolic acid, unsaturated fatty acids, and other active substances, have been recorded in sea buckthorn, many of which are now being analyzed in detail for their medicinal properties [5,6]). Commercial products promoted as remedies for various illnesses, including cosmetics for sensitive skin treatment, are usually based on dried fruits, fruit pulp oils, and/or seed oils. These products are often manufactured as, e.g., fruit powders, oil capsules, or vitamin C tablets.

In some countries, especially in Europe and North America, sea buckthorn is highly appreciated for its tasty fruits that are used in the food industry to manufacture various culinary products like juice, wine, liquor, syrup, cakes and pies, curd, jam, candy, and ice cream [3]. The taste is tart and quite unique, often requiring considerable amounts of sweetening to produce a balanced flavor. A need for inherently sweeter fruits has therefore been addressed in some plant breeding programs.

## 2. Taxonomy

The genus *Hippophae* contains several diploid (2n = 24) taxa, but until now, only the most widespread and variable species, *H. rhamnoides*, has been domesticated. Nevertheless, other species, all of which are restricted to cold-temperate areas on the Qinghai–Tibet plateau and adjacent areas in Central Asia, are being investigated for valuable traits to be exploited in the future.

Despite the relatively small number of taxa, systematic treatment of the genus *Hippophae* has been quite controversial, and several treatises have been published [7]. Rousi [8] recognized three species: *H. rhamnoides*, *Hippophae salicifolia* D. Don, and *Hippophae tibetana* Schlecht., and up to nine subspecies of *H. rhamnoides*. Subsequently, several additional species and at least one subspecies have been described and published: *Hippophae goniocarpa* YS Lian, XL Chen and K Sun ex Swenson and Bartish; *Hippophae gyantsensis* (Rousi) YS Lian; *Hippophae litangensis* YS Lian and XL Chen ex Swenson and Bartish; *H. neurocarpa* SW Liu and TN He; and *Hippophae neurocarpa* subsp. *stellatopilosa* YS Lian, XL Chen and K Sun ex Swenson and Bartish.

In 2000, the first molecular marker-based treatise of the entire genus *Hippophae* (15 taxa) was presented using Random Amplified Polymorphic DNA (RAPD) markers [9]. Other RAPD-based studies have focused on a subset of the taxa, e.g., *H. rhamnoides* subsp. *sinensis* and *H. rhamnoides* subsp. *mongolica* [10] and *H. rhamnoides* subsp. *sinensis* and other Chinese taxa [11]. Subsequent investigations have been undertaken using, e.g., chloroplast DNA (cpDNA) and morphological characters [12] as well as Internal Transcribed Spacer (ITS) sequences [13]. Other molecular methods like Simple Sequence Repeats (SSR) markers [14] have been applied to investigate relationships among the different taxa of *Hippophae*, while Amplified Fragment Length Polymorphism (AFLP), Selective Amplification of Microsatellite Polymorphic Loci (SAMPL) [15], and DNA barcode markers (ITS, matK, rbcL, and rpoC1) [16] have been applied to *H. rhamnoides* subsp. *turkestanica*, *H. salicifolia*, and *H. tibetana* in northern India and the Himalayas.

Two sections, sect. *Gyantsenses* (*H. gyantsensis*, *H. tibetana*, and *H. neurocarpa*) and sect. *Hippophae* (*H. salicifolia* and *H. rhamnoides*), have been recognized based on whether the carpodermis is fused with the seed coat or not. A recent analysis based on multiple chloroplast and nuclear gene fragments has instead shown that *H. gyantsensis*, *H. neurocarpa*, and *H. salicifolia* form one clade, whereas *H. tibetana* forms a second clade together with *H. rhamnoides* [1]. In addition, three species appear to be hybridogenic: *H. gyantsensis* has probably derived through gene flow between *H. rhamnoides* subsp. *yunnanensis* and *H. neurocarpa*, whereas *H. goniocarpa* derives through gene flow between *H. rhamnoides* subsp. *sinensis* and *H. neurocarpa*, and *H. litangensis* through gene flow between *H. rhamnoides* subsp. *sinensis* and *H. neurocarpa* subsp. *stellatopilosa* [12,17,18].

The most variable and economically important species, *H. rhamnoides*, is generally divided into five subspecies in Asia: subsp. *caucasica* Rousi, subsp. *mongolica* Rousi, subsp. *turkestanica* Rousi, subsp. *sinensis* Rousi, and subsp. *yunnanensis* Rousi, and three in Europe: subsp. *rhamnoides* L., subsp. *fluviatilis* (Soest) Rousi, and subsp. *carpatica* Rousi. Most populations are found mainly on seashores and river deltas or on valley slopes up to 5000 m above sea level, i.e., in habitats typical of early successional species. The first taxon to be cultivated was subsp. *mongolica*, which has been utilized for almost a century in Siberia, but subspecies *sinensis*, *turkestanica*, *rhamnoides*, *carpatica*, and *caucasica* have also been used in cultivation and plant breeding.

Since the phytochemical contents differ considerably among the subspecies of *H. rhamnoides*, efforts have been made to develop taxon-specific molecular markers that can detect adulteration and contamination in commercial fruits and derived products. A high-resolution melting assay based on a DNA barcoding region of ITS2 in the ribosomal DNA (rDNA) could discriminate among all seven *Hippophae* species [19]. Single Nucleotide Polymorhism (SNP) markers from the 45S rDNA region were later used to distinguish subsp. *mongolica* and subsp. *sinensis* and apparently produce reliable results even with very low DNA concentrations [20].

## 3. Population Genetics

Information on genetic diversity is very helpful when collecting and utilizing plant material for gene banks and plant breeding. Lately, DNA-based markers have been used to assess the amount and partitioning of genetic variability in numerous plant species. Considering the life history traits of *H. rhamnoides*, which is a perennial (wild plants generally live for 30–60 years), obligately outcrossing, wind-pollinated, early successional species with seed dispersal through birds, relatively high genetic variation is expected within populations, together with low differentiation between populations [21].

A RAPD-based study carried out on 10 populations of *H. rhamnoides* in Northern Europe showed that within-population genetic variation, estimated as expected heterozygosity H_E_, was on average 0.16 [22]. This value is slightly lower than the mean value of 0.22 reported in a metastudy with 60 RAPD-based studies of different species [21]. Analyses of the impact of various life history traits showed that breeding system and successional status are highly important; outcrossing species had the highest within-population diversity (0.27 on average), while early successional species instead had the lowest (0.17 on average).

Analysis of Molecular Variation (AMOVA) allows the partitioning of genetic variation between and within populations. For sea buckthorn, only 15% of the variation occurred between populations, which is considerably lower than the mean value of 34% in 116 studies [21]. This parameter is affected by the breeding system (lowest values for outcrossing species), successional status (highest values for early and medium-early species), life form (lowest values for long-lived perennials), and seed dispersal (lowest values for species with animal-ingested seeds). Apart from the early successional status of *H. rhamnoides*, the other life history traits are in keeping with low genetic differentiation between populations.

In another RAPD-based study, 13 populations of *H. rhamnoides* subsp. *sinensis* were found to have rather low average within-population diversity = 0.17, as well as restricted population differentiation = 18 [23]. In yet another study, Inter Simple Sequence Repeat (ISSR) markers were applied to 11 sea buckthorn populations in Northeastern and Northwestern China [24]. Within-population diversity was in keeping with the other studies, ranging from 0.16 to 0.21, but population differentiation was only 7%. A second ISSR study on 15 Chinese populations yielded an average within-population diversity of 0.20 for 7 populations of subsp. *yunnanensis*, 0.22 for 7 populations of subsp. *sinensis*, and 0.14 for the single population of subsp. *gyantsensis* [25]. Overall differentiation among all 15 populations was 14.5%, and a cluster analysis showed that the subsp. *gyantsensis* population differed considerably from the others in accordance with a proposed species status as *H. gyantsensis*.

SSR markers were applied to distinguish between *H. rhamnoides* subsp. *sinensis* and *H. rhamnoides* subsp. *yunnanensis* and to estimate the genetic diversity within each taxon [26]. The 32 investigated populations could be grouped according to taxonomic status, with indications of some inter-subspecies hybridization in the zone of partly overlapping distribution. Populations of subsp. *sinensis* were more variable, with observed heterozygosity (*H_o_* = 0.40) compared to subsp. *yunnanensis* (*H_o_* = 0.20), whereas population differentiation was 26% and 37%, respectively, in these two subspecies. Both within-population diversity estimates are somewhat low compared to an average *H_o_* = 0.58 in 80 SSR-based studies, whereas between-population estimates were more similar to the average of 24–26% in 51 studies [21].

Most studies have failed to find an association between genetic and geographic distances in sea buckthorn [12,23,25]. Within-population variation appears to be especially high in species and subspecies that occur close to the center of origin in Central Asia, e.g., *H. tibetana*, and in the possibly hybridogenous species *H. goniocarpa* [9]. By contrast, small and isolated populations (e.g., growing at high altitudes) often appear to be more homogenous [27,28]. For a more comprehensive review of population genetics and phylogeography in *Hippophae*, see Bartish et al. [7].

## 4. Sex Determination

In sea buckthorn, sex is genetically determined through an X/Y system and heteromorphic sex chromosomes [29], with the Y-chromosome being slightly longer than the X-chromosome. In commercial sea buckthorn orchards, the majority of plants are female, but with the addition of about 10% male plants to ensure adequate pollination. Plant breeding is therefore mainly directed towards the development of high-quality female plants. Male seedlings are often discarded as soon as their gender can be ascertained, but morphology-based gender determination cannot be undertaken until the plants flower for the first time, which usually takes 3–5 years. A method for early discrimination between male and female seedlings would save much time and space in plant breeding programs as well as in seedling-based plantations.

Genes related to sex have been identified in several dioecious plants, but in, e.g., papaya (*Carica papaya* L.), factors such as the environment, hormones, and genetic and epigenetic background can apparently also affect sex expression [30]. Nevertheless, molecular markers have been developed and are now being successfully used for early sex determination in papaya and other crops.

Attempts have also been made to develop gender-specific DNA markers in sea buckthorn, but these have usually not been sufficiently consistent when screened in a more diverse germplasm. The first attempt was based on RAPD analysis of offspring derived from experimental crosses in *H. rhamnoides* subsp. *rhamnoides* [31]. A male-specific DNA marker was found using Bulked Segregant Analysis (BSA), but when applied to individual plants, this marker worked only for the progeny obtained in one of the two tested crosses. Several RAPD-based studies have also been carried out on *H. rhamnoides* subsp. *turkestanica* in northern India. One male-specific marker was found but validated in only five plants of each sex [32]. In another study, two female-specific markers were found and subsequently converted into Sequence-Characterized Amplified Region (SCAR) markers [33]. These were validated in a larger material but derived from only one population. A female-specific RAPD-based marker was developed for subsp. *sinensis* and validated in more diverse material [34].

An ISSR-derived male-specific band was converted into a SCAR marker and validated in subsp. *turkestanica* plants from three geographically isolated valleys in the Ladakh region of India [35]. In a broader approach, RAPD, ISSR, SSR, and MADS box gene-specific markers were applied, with one of the RAPD primers producing two male-specific fragments [36]. These could, however, discriminate between males and females in only one of the populations when screened on material obtained from both the Ladakh region (Jammu and Kashmir) as well as Lahaul and Spiti, and Kannaur (Himachal Pradesh).

In the last two decades, transcriptomic analyses have revealed important information about gene action in many plant species. This approach has also recently been employed in sea buckthorn to search for gender-specific genes. Thus, 21 floral regulatory genes, homologous to previously established sequences in model plant species, proved to be differentially expressed across the developmental stages of male and female flowers in samples of *H. rhamnoides* [37]. Two possibly promising genes were identified: *HrCRY2* (cryptochrome receptor gene) was significantly over-expressed in female flowers, whereas *HrCO* (circadian pathway gene) was significantly over-expressed in male flowers. Further research is still needed to determine the role of these genes in the development of male and female flowers.

The observed difficulties in developing robust gender-specific markers may be connected to the low differentiation between the male and female genomes in *Hippophae*. A survey of 25 wild populations of *H. rhamnoides* subsp. *turkestanica* in northern India showed that 2–4% of the plants were polygamomonoecious (PGM) with male, female, and hermaphrodite flowers, suggesting that the transition from hermaphrodites (the original state for angiosperms) to dioecy (the derived state) happened recently in this genus [38]. Moreover, the male and female genomes appear to be very similar overall. A Representational Difference Analysis (RDA) of DNA sequences was applied to the search for gender-specific differences between male and female genomes. Some of the obtained polymorphisms looked promising initially, but none of them held up when screened on plants from more distant populations [38].

An AFLP analysis conducted on five populations of subsp. *turkestanica* was more successful and produced 4 female-specific and 2 male-specific fragments that were cloned and sequenced and subsequently used for developing SCAR primers [38]. The genomic region around each SCAR was explored through genome walking, and new primers were designed and then tested on 50 male and 50 female plants. Most of the tested regions were not informative when applied to a more diverse plant material, but with one exception: primers for the locus *HRML* (*H. rhamnoides* male locus) produced a 329 bp fragment in all male plants tested in 25 populations. This has been sequenced and further characterized, and it is now available as a 7 kb region named HRMSSR in NCBI Genbank accession KX444194 [38].

While most of the research has been carried out on *H. rhamnoides*, a similar sex determination system is probably also present in the other species. A RAPD-based female-specific marker was thus found in *H. goniocarpa* [39]. In a more recent study, 80 *H. tibetana* samples were sampled from four sites with 10 male and 10 female plants in each site and screened for male-specific markers using a comparative analysis of Restriction-Associated DNA Sequencing (RAD-seq) data [40]. A Genome-Wide Association Study (GWAS) indicated that several SNPs on Chromosome 2 are related to male sex determination. Much additional work is, however, needed to determine the location of sequences that can be used for the development of sex-specific markers.

It should also be mentioned that the occurrence of facultative apomixis, i.e., seed set without pollination, has been reported in *H. rhamnoides* based on some seed setting in bagged inflorescenses (16% fruit set as compared to 68% fruit set in unbagged inflorescenses) and on cytological evidence of both apospory and adventive (nucellar) embryony in embryo sacs and in fruits obtained after bagging, respectively [41]. The studied plant material was collected in northern India and presumably belongs to subsp. *turkestanica*. In a follow-up study on five wild sea buckthorn populations in India, only a 3% fruit set was recorded in the bagged inflorescenses, compared to a 60% fruit set in the unbagged inflorescenses [42]. This strong reduction in fruit set after bagging is not typical of apomicts in general, and the extent to which apomixis plays a role in the genus requires more research. The effects of apomixis on the genetic variability of seedling offspring after natural pollination or after the undertaking of experimental crosses have also not yet been reported to our knowledge.

## 5. DNA-Based Identification of Cultivars

**Figure 2:**
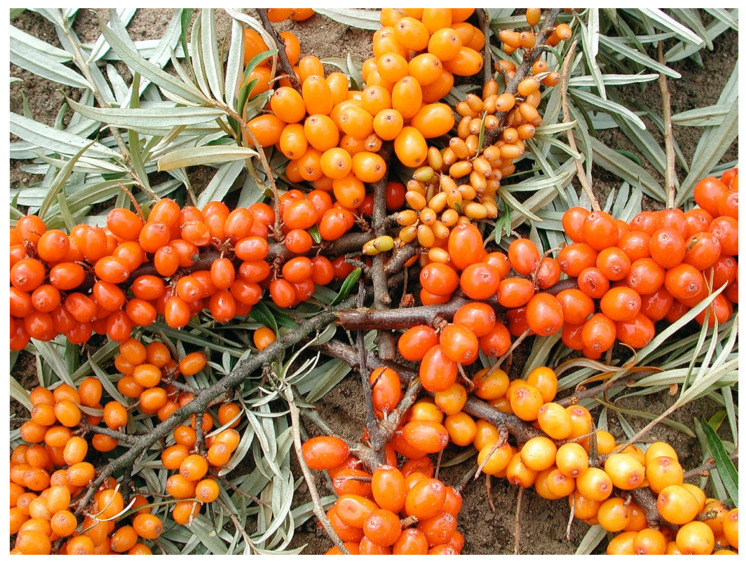
Variation among sea buckthorn (*Hippophae rhamnoides*) genotypes.

Sea buckthorn cultivars vary widely in, e.g., fruit size, color and shape, but are still often difficult to identify properly based on only morphological traits (Figure 2). Accessions of the same cultivar can also vary to an unexpected extent when grown under different conditions that affect, e.g., fruit quality and levels of resistance. Methods are therefore needed for the unambiguous identification of germplasm (wild material, lines, or elite cultivars). Presently, this can be achieved through the application of various types of DNA markers. Since dioecious reproduction results in obligate outcrossing, all seedlings have unique genotypes even when obtained from the same seed parent. Moreover, sea buckthorn cultivars and advanced selections are propagated vegetatively and are therefore expected to consist of a single genotype that can be identified and distinguished from all other cultivars and selections with DNA fingerprinting.

Almost any type of molecular marker method that produces a sufficient number of data points, like RAPD, ISSR, and AFLP, will usually allow discrimination between different plant accessions [3]. For the setting up of joint international databases, SSR markers and SNP data are, however, usually preferred since results from different laboratories can be entered and compared as long as the same set of SSR primers or the same SNP arrays are used. Such databases are now developed to an increasing extent in many crops and are used as a basis for defining cultivar-specific profiles like the SNP-based Malus UNiQue genotype (MUNQ) codes in apple [43] and the SSR-based Cherry UNiQue genotype (CHUNQ) codes in cherry [44]. So far, joint international databases have not yet been developed for sea buckthorn, making it difficult to evaluate data across different studies.

The first major screening of cultivated sea buckthorn germplasm was performed on 55 accessions in a Swedish gene bank using RAPD [45]. Approximately 20 accessions derived from crosses between subsp. *mongolica* and subsp. *rhamnoides*, 15 belonged to subsp. *mongolica*, 10 belonged to subsp. *rhamnoides*, and the remainder represented either subsp. *fluviatilis*, subsp. *carpatica*, subsp. *caucasica*, or other crosses. Cluster and Principal Coordinate (PCO) analyses showed considerable grouping in accordance with the taxonomic and geographic origination.

Efficient discrimination among genotypes and grouping according to origin has been reported in other RAPD-based studies on the different subspecies of *H. rhamnoides* with wild as well as cultivated material [10,46,47,48]. Similar results have also been obtained when using other types of multilocus markers, like ISSR [49] and AFLP [50].

For the development of more robust databases, SSR loci are often preferred. The first report in sea buckthorn described primers for nine genomic SSR (gSSR) loci [51], and was soon followed by primers using expressed sequence tags (estSSR) [52]. Numerous analyses have since then been undertaken in different sets of sea buckthorn material using SSR loci that generally seem to be transferable across all taxa in the genus *Hippophae* [14,53,54,55,56,57,58].

Regardless of the marker system used, the grouping of the accessions has in general reflected the taxonomic classification into different subspecies as well as the geographic origin of the accessions themselves or the breeding program where they were developed. Information has been obtained about the origin of named cultivars, either as selections from local wild populations or as crosses in breeding programs. Moreover, the application of DNA markers to germplasm collections has frequently proved useful in detecting labeling problems like the presence of more than one name for the same cultivar or different cultivars sharing the same name.

## 6. Sea Buckthorn Breeding around the World

Sea buckthorn fruits are still harvested from wild or naturalized stands in many countries in Asia and Europe, but the increasing interest in this crop has boosted the establishment of commercial orchards. Since most orchards are managed as low-input and/or organically, i.e., without fungicides or pesticides, a healthy plant material is required. Low-cost orchards are sometimes planted with seedlings, but the utilization of genetically superior and vegetatively propagated cultivars holds more potential in the long run (Table 1). Consequently, a development in the derivation of suitable plant material can be seen, from (1) selection among wild plants or their offspring, to (2) selection among offspring after open pollination of superior genotypes, and to (3) selection among offspring derived from crosses between selected parents.

Conventional breeding programs for improving adaptability, tolerance, yield, and quality of sea buckthorn have been undertaken since the early 1900s, and more than 150 cultivars have been developed for culinary and/or medicinal use [79]. Breeding was initiated in Russia in the 1930s and in China in 1985 [61,80] using *H. rhamnoides* subsp. *mongolica* from the Altai mountains, and subsp. *sinensis* in Northern China, respectively. Cultivars based on subsp. *sinensis* are usually adapted to harsh environments, grow fast, and have a very high vitamin C content, but their major drawbacks are very thorny branches, low fruit yield, small fruits, and high acidity. By contrast, cultivars based on subsp. *mongolica* often have fewer thorns, a high fruit yield (5–12 kg/plant), large fruits (30–120 g/100 fruits), high oil content, and less acidity. Unfortunately, these cultivars are less well adapted to high summer temperatures and drought and are more susceptible to fungal diseases and pests. The third major subspecies to be used in plant breeding is subsp. *rhamnoides*, which in many respects takes an intermediate position compared to subsp. *sinensis* and subsp. *mongolica*. Russia (over 70 cultivars) and China (over 60 cultivars) are still the leading countries in sea buckthorn improvement, and many cultivars from these countries are planted around the world.

**Figure 3:**
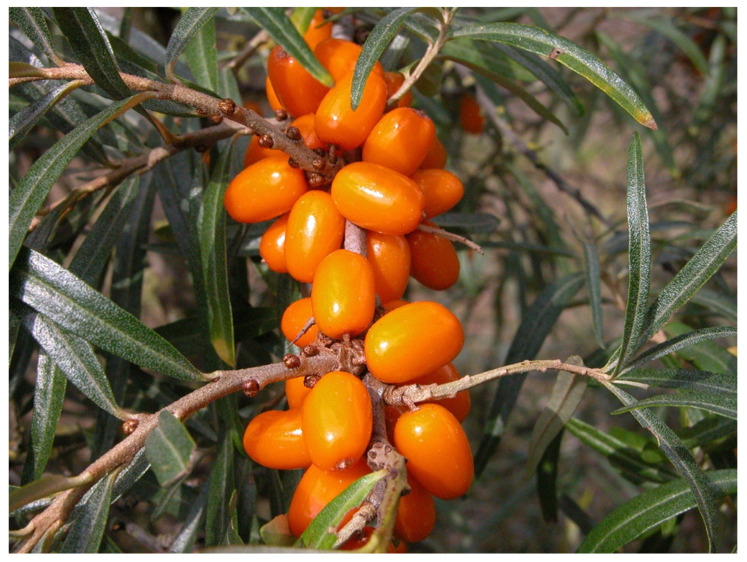
‘Botanicheskaya Ljubitelskaya’, Russian cultivar (*Hippophae rhamnoides* subsp. *rhamnoides* × subsp. *mongolica*).

In Russia, selection and breeding have been carried out in different locations, from the maritime climate in Leningrad to a more continental climate in Moscow, where selection and breeding were initiated in 1952 [81]. The domestication, however, started further east, in Barnaul in the Altai region of Siberia. Three cultivars (‘Dar Katuni’, ‘Novost Altaya’, and ‘Zolotoy Pochatok’) derived by selection in wild stands from Katun (Gomy Alta) were released in the 1960s by the Lisavenko Research Institute of Horticulture for Siberia [61]. Accessions were also collected and evaluated from a wider geographic area, and additional cultivars were released, like ‘Maslichnaya’, ‘Vitaminnaya’, ‘Oranzhevaya’, and ‘Chuyskaya’. Later, well-known cultivars were also developed at other locations in Russia, from crosses between different accessions of subsp. *mongolica* as well as from crosses between subsp. *mongolica* and subsp. *rhamnoides* like, e.g., ‘Botanicheskaya Ljubitelskaya’ (Figure 3) and ‘Trofimovskaya’ developed in Moscow. Some breeding has also taken place in Mongolia, resulting in, e.g., ‘Ulaangom’ and ‘Chandman’.

**Figure 4:**
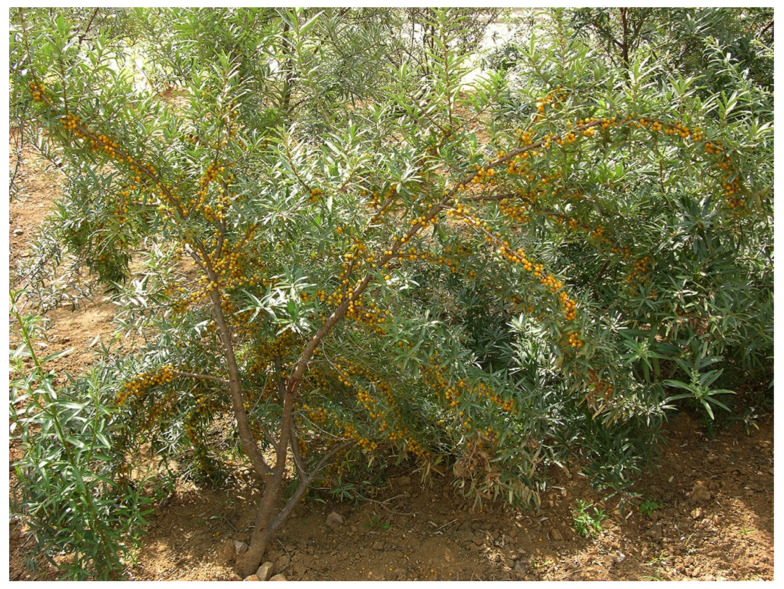
Plant of *Hippophae rhamnoides* subsp. *sinensis*.

**Figure 5:**
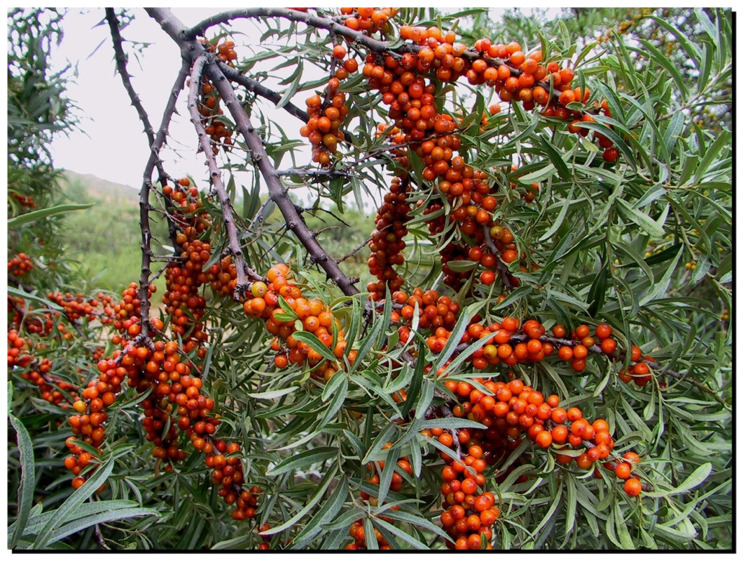
‘Mengzhonghong’, Chinese cultivar (*Hippophae rhamnoides* subsp. *mongolica* × subsp. *sinensis*).

In China, breeding and selection were initially based on the indigenus subsp. *sinensis* (Figure 4). Five cultivars, including ‘Hongxia’ and ‘Wucixiong’ (male), were obtained through the selection of open-pollinated offspring from local stands [69]. Another 15 cultivars, including ‘Wulanshalin’, ‘Hunjin’, and ‘Wucifeng’ that have no or few thorns, large fruits and long fruit stalks, and yields of 10,000–20,000 kg/ha, were selected among offspring from introduced sea buckthorn accessions from Mongolia and Russia (subsp. *mongolica*) [70,71]. The hardy and highly drought-resistant cultivar ‘Qiuyang’ was selected among seedlings derived from open-pollination of the subsp. *mongolica* cultivar ‘Wulangmu’ [75]. This step in the domestication process was then followed by making controlled crosses between subsp. *sinensis* and subsp. *mongolica* to improve adaptability, yield, and quality [80]. About 20 cultivars, like ‘Hualin 1′, ‘Mengzhonghuang’, ‘Mengzhonghong’ (Figure 5), ‘Dalate’, ‘Ezhonghuang’, ‘Ezhongxian’, ‘Chengse’, ‘Hongyun’, ‘Hongji 1′, ‘Zhongji 3′, and ‘Zhongji 4′, have now been released based on these crosses [63]. Some other new cultivars, such as ‘Gaoyou 1′, ‘Chaoyang’, ‘Wanxia’, and ‘Wanhuang’, have also been released recently in China.

Many other Asian countries have now initiated their own programs for developing sea buckthorn as a crop plant. Harvesting has initially been conducted in native stands of, e.g., subsp. *turkestanica* (India) or subsp. *caucasica* (Iran and Turkey), but these are in general unsuited for large-scale commercial plantations due to thorniness, small fruit size, and low fruit yield. In India, evaluation and selection are presently undertaken in locally grown offspring obtained from the seeds of Russian subsp. *mongolica* cultivars [82], and a similar development is also seen in other Asian countries.

In Ukraine, plant breeding has resulted in cultivars like ‘Lvivyanka’, ‘Osinnia krasunia’, ‘Mukshanska’, ‘Rapsodiia’, and ‘Medova osin’ [73]. The first four of these cultivars are based on crosses involving subsp. *mongolica* and produce larger fruits and higher yields compared to the fifth, which is derived from subsp. *carpatica* and instead is characterized by high winter hardiness, drought tolerance, and disease resistance. Some cultivars have also been developed in neighboring Belarus, including the productive ‘Plamennaya’ with fruits of 80 g/100 fruits [61]. Commercial production is also undertaken in, e.g., Romania and, recently, Greece, but is mostly based on Russian cultivars derived from subsp. *mongolica*.

In Berlin, in Germany, cultivars like ‘Askola’, ‘Dorana’, ‘Frugana’, ‘Hergo’, and ‘Leikora’ were released from 1979 onwards, based on selection in wild stands of subsp. *rhamnoides* [60]. Increased focus on oil content prompted a second step in the breeding program, with renewed selection activities in subsp. *rhamnoides* as well as the undertaking of crosses with subsp. *mongolica* from the Altai region, resulting in ‘Sirola’ and ‘Habego’ (‘Orange Energy’).

**Figure 6:**
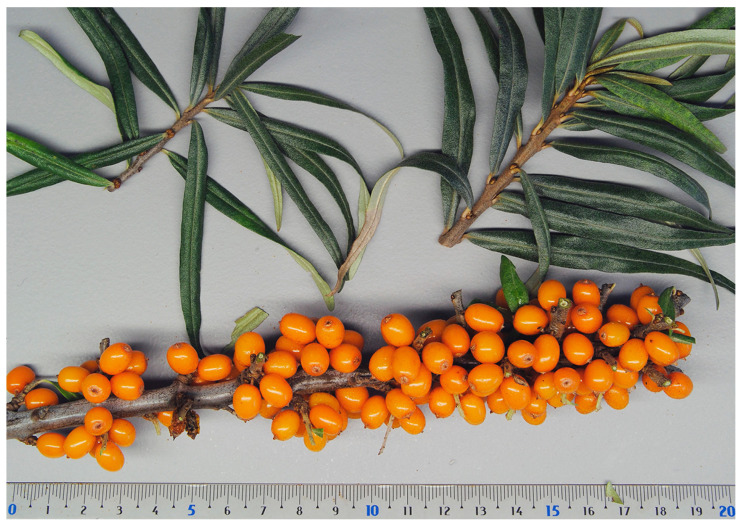
‘Raisa’, Finnish cultivar (*Hippophae rhamnoides* subsp. *rhamnoides* × subsp. *caucasica*).

**Figure 7:**
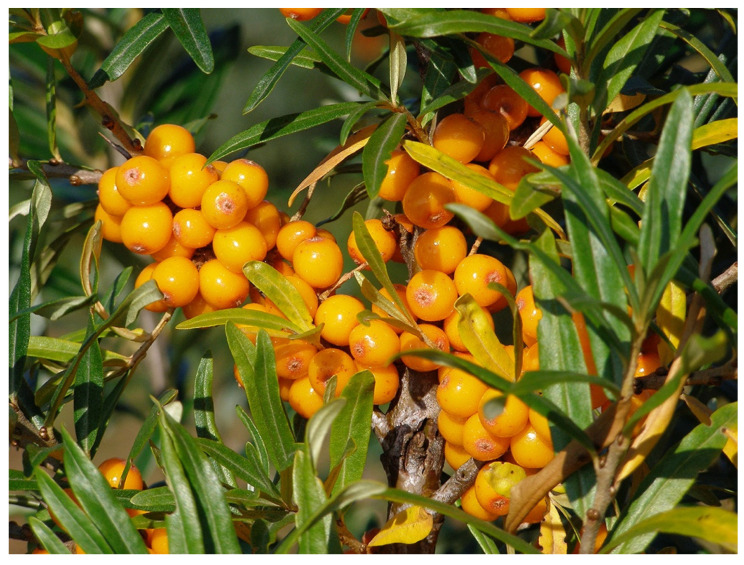
‘Sol’, Swedish cultivar (*Hippophae rhamnoides* subsp. *rhamnoides* × subsp. *mongolica*).

Domestication of sea buckthorn in the Nordic and Baltic countries (mainly Sweden, Finland, Estonia, and Latvia) was influenced by both Germany and Russia. Some of the German cultivars were not sufficiently cold-hardy to ensure survival in Finland and the Baltic countries [67] and ripened too late. In Finland, ‘Terhi’ and ‘Tytti’ were obtained from native stands of subsp. *rhamnoides* [76], while ’Raisa’ was derived from a cross between subsp. *rhamnoides* and subsp. *caucasica* (Figure 6). Nowadays, breeding relies mainly on crosses using subsp. *rhamnoides* or subsp. *fluviatilis* as one parent and subsp. *mongolica* as the other parent. Production of juice is a major goal, and the hybrid cultivars have larger fruits, reduced acidity, perceived sweeter juice, superior juicing capacity (80–90% compared to 50–60% in subsp. *rhamnoides*), and significantly fewer troublesome stellate hairs on the fruits. Hybrid cultivars developed in Sweden, where a breeding program started in 1986, are, e.g., ‘Julia’, ‘Lotta’, ‘Sol’ (Figure 7), ‘Idun’, ‘Eir’, ‘Fenja’, and ‘Torun’, and in Latvia, e.g., ‘Mary’ (‘Marija Bruvele’) and ‘Tatjana’ [54].

In Canada, a breeding program was initiated in 1995, resulting in, e.g., ‘Harvest Moon’ derived from open pollination of a subsp. *mongolica* seedling obtained from Siberia. This early-maturing, relatively thornless, and winter-hardy cultivar has large reddish-orange fruits with long pedicels, which facilitate hand harvesting and is well suited for growing in the Canadian Prairies and northern Great Plains of the USA [66].

In the future, transgenesis and genome editing with Clustered Regularly Interspaced Short Palindromic Repeats-associated Protein 9 (CRISPR/Cas9) may become useful for improving commercially important traits in sea buckthorn. Still, the notion of using gene transfer or gene editing methods may prove unacceptable for a crop destined for the health food market. Since only a few protocols are available for regeneration [83] and transformation of sea buckthorn [84], a lack of efficient regeneration protocols could also hamper the application of these methods.

## 7. Plant Breeding Goals

In order to maximize the economic, ecological, and social benefits of sea buckthorn cultivation, breeding goals have focused on (1) enhancing the yield of fruits and seeds, (2) improving the contents and quality of oils and bioactive components, and (3) increasing the tolerance to extreme temperatures (both high and low), drought, waterlogging, diseases, and pests.

### 7.1. Plant Architecture and Yield

The small and inconspicuous flowers are borne in tight clusters on 2-year-old branches. It takes approximately 100–120 days from fertilization to ripe fruits. Yield is influenced by several factors like plant growth, habit and shape, internode length, fruit size, and pedicel length; phenological events like flowering period and time of fruit maturity; and physiological traits such as disease resistance and tolerance to stress.

The ideotype for sea buckthorn plant shape depends on the harvesting method, which usually consists of hand-picking in the field, direct juicing in the field, cutting off whole branches, which are then frozen and threshed, or machine harvesting in the field [85]. For hand-picking and juicing in the field, a rather short plant with as few thorns as possible is desirable. Thorniness does not matter that much if whole branches are cut off, but longer fruit stalks (5–10 mm) allow easier detachment of fruits. Plant size and ripening time can also be important depending on the type of branch cutting applied (e.g., total cut, lower cut, vertical split, and horizontal split [86]). The machine harvesting of sea buckthorns is conducted on a restricted scale, usually with either vacuum or vibrations; both types of harvesters work better on cultivars with long fruit stalks, and the latter type also requires branches that bend easily without breaking.

Late-ripening plants are generally expected to have higher yields than early-ripening plants since their period of active growth and development is longer. Very-late-ripening plants are, however, vulnerable to early periods of freezing before full ripening. In high-latitude regions, late-ripening individuals may also fail to form sufficient flower primordia for the following year [87].

The fruit of sea buckthorn is not really a berry and is better described as a ”pseudodrupe” [41]. Fruit size and shape are commercially important traits that are determined by several processes, including cell division and cell expansion. Interspecific variation in these processes has been demonstrated for three species of *Hippophae* [88], but this information is not yet immediately applicable in applied plant breeding.

Generally, cultivars derived from subsp. *mongolica* have the largest fruits, like the Siberian ‘Elizaveta’, ‘Agurnaya’, and ‘Avgustina’, which are reported to produce 100–120 g/100 fruits when grown in Russia [61]. In a study of 78 accessions in Northern China, cultivars of subsp. *sinensis* had much smaller fruits (average of 11 g/100 fruits) compared to subsp. *mongolica* (average of 48 g/100 fruits), while hybrids took an intermediate position (average of 31 g/100 fruits) [58]. Cultivars based on subsp. *caucasica* also tend to produce small fruits, as reported for 10 accessions in Eastern Anatolia, where weight ranged from 14 to 24 g/100 fruits [89]. One subsp. *carpatica* cultivar in Ukraine had larger fruits, 37 g/100 fruits, while four hybrid cultivars (subsp. *mongolica* × subsp. *carpatica*) varied from 63 to 71 g/100 fruits, i.e., similar to pure subsp. *mongolica* (68 g/100 g) [73].

### 7.2. Resistance against Abiotic Dress

Cold tolerance: Plants of *H. rhamnoides* have been reported to tolerate temperatures between −43 °C and +40 °C but grow best when the average temperature in the hottest month ranges from 15 to 25 °C [90]. There is considerable variation in cold hardiness between plants from different geographic origins. Both subsp. *mongolica* and subsp. *sinensis* are adapted to a continental climate and can tolerate very cold winters. In a cool maritime or semi-maritime climate with fluctuating winter temperatures, as in Northern Europe, imported cultivars of subsp. *mongolica* suffer considerably more winter injury compared to native plants of subsp. *rhamnoides*. Several inter-subspecies hybrids originally bred in Russia (especially Moscow) instead have satisfactory cold hardiness when grown in, e.g., Finland, Sweden, Estonia, and Latvia [54,91], and similar crosses are now undertaken in these countries. *H. tibetana*, which grows in alpine plateau regions, can tolerate very low temperatures during the growing season but has not yet been used in breeding programs.

Variation in cold hardiness among genotypes of various origins is, to a large extent, affected by their biochemical content. Cold acclimation increases the concentrations of sugars and dehydrins, resulting in elevated freezing tolerance. Levels of dehydrin mRNA as well as sugar components (especially sucrose) appear to correlate with the cold hardiness of the leaves [87]. Further analyses of the biochemistry of cold hardiness may result in the identification of key genes and the development of DNA markers for application in plant breeding. Large phenotyping projects are, however, needed to supply information about allelic variation in these genes. Thus, data on carefully evaluated parameters involved in cold hardiness must be collected for a large set of genotypes.

Drought tolerance: Although sea buckthorn plants can endure lengthy periods of drought better than many other woody shrubs, flowering and fruit set are adversely affected by inadequate soil moisture, particularly during early spring. Irrigation may therefore be required for orchards in areas with low precipitation. Since this can be difficult and costly to implement, naturally high levels of drought tolerance would be beneficial for cultivars grown in dry areas.

There is, as of yet, little information on genetic variation in parameters involved in water uptake and use. Research on drought stress has shown that leaf water potential, photosynthetic rate, and stomatal conductance decrease in genotypes of both subsp. *sinensis* and subsp. *mongolica*, whereas the content of polyphenols such as flavone, flavonol, isoflavone, and flavanone decreases only in subsp. *mongolica* [92]. By contrast, flavone and abscisic acid (ABA) contents were significantly higher in subsp. *sinensis*. In a transcriptomics analysis, numerous Differentially Expressed Genes (DEGs) were identified under drought stress in the two genotypes [92]. These DEGs were associated mainly with carotenoid biosynthesis, flavonoid biosynthesis, photosynthesis, and plant hormone signal transduction. Six hub DEGs, which play a role in the ABA-dependent signaling pathway, were identified. The mutual regulation of ABA and flavonoid signaling apparently contributes to the difference in drought resistance between the two analyzed samples. Any kind of stress is known to activate plant-specific transcription factor *TCP* genes. In sea buckthorn, *HrTCP20* was significantly up-regulated under drought stress, suggesting that this *TCP* has a role in drought tolerance, possibly by affecting the biosynthesis of the important plant hormone jasmonic acid [93].

### 7.3. Resistance against Fungal Diseases

Fungal diseases have become increasingly problematic in sea buckthorn, causing serious damage in both wild stands and commercial orchards all over the world [94]. Unfortunately, there is a lack of in-depth pathological studies with bioassays to quantify the symptoms caused by unambiguously identified fungi. Commonly observed symptoms in the field after natural infection are various cankers and lesions on the stems of trunks and main branches, wilting leaves and buds, followed by necrosis of both shoots and roots, and eventual death of the entire plant. In addition, several fungi have been reported to affect leaves and/or fruits, but these symptoms are seldom quite as serious as the three diseases described below.

Wilt: Drying up of sea buckthorn plants, also known as wilt, is a serious worldwide problem. Soil-borne fungi belonging to the genera *Fusarium* (several species) and *Verticillium* (*Verticillium dahliae* and *Verticillium albo-atrum*) have been identified in connection with wilt in Europe, including Russia, as well as in India and Canada. Early symptoms vary between orchards in different countries and are possibly associated with the origin of the planted material but also with environmental factors like drought and excessive soil moisture. Thus, Russian cultivars, presumably derived from subsp. *mongolica*, were reported to be more sensitive to *Verticillium* in Germany in comparison with other cultivars [67]. Cultivars with above-average tolerance to wilt in a field trial with 42 cultivars and advanced selections in Belarus include ‘Desert maslichnyi’, ‘Yolochka’, ‘Mendeleevskaya’, ‘Syurpriz Baltiki’, and ‘Zolotoi klyuchik’ [62].

Stem canker: Several fungi, e.g., *Hymenopleella hippophaeicola*, *Cytospora* spp., and *Stigmina* spp., have been reported to cause stem canker and leaf spot on sea buckthorn around the world. The symptoms can lead to the death of entire shoots and may eventually kill the whole plant. In Finland, Russian cultivars derived from subsp. *mongolica* appear to be especially susceptible to damage by *Stigmina*, whereas wild stands of subsp. *rhamnoides* remain healthy [95]. Tolerance to *Stigmina* seems to depend on the ability to withstand frost damage in spite of the fluctuating temperatures during autumn typical of a coastal climate.

Dried shrink disease: The concept of Dried Shrink Disease (DSD) was introduced to denote a common infection pattern caused by various fungi in China but has also been reported in, e.g., Sweden and Russia [3]. DSD has been a major factor in limiting the success of sea buckthorn plantings in China for several decades. Leaves of infected plants become increasingly chlorotic at the beginning of summer, and branches bend down, wilt, and eventually dry up. Fruits of infected plants do not develop properly, and many drop well before maturation. Possibly, wilt and stem canker, as described above, are part of the DSD concept, but other symptoms also occur. In addition to *Fusarium acuminatum*, *Fusarium oxysporum*, *Fusarium camptoceras*, *Stigmina*, and *Verticillium*, fungi that have been reported in connection with DSD include *Phomopsis* (=*Diaporthe*), *Phellinus hippophaeicola*, and *Dothidea hippophaes* (=*Plowrightia hippophaes*) [3,96]. *Hymenopleella* and *Diaporthe* were the most frequently found species in symptomatic samples in Germany [97].

### 7.4. Resistance against Insects

**Figure 8:**
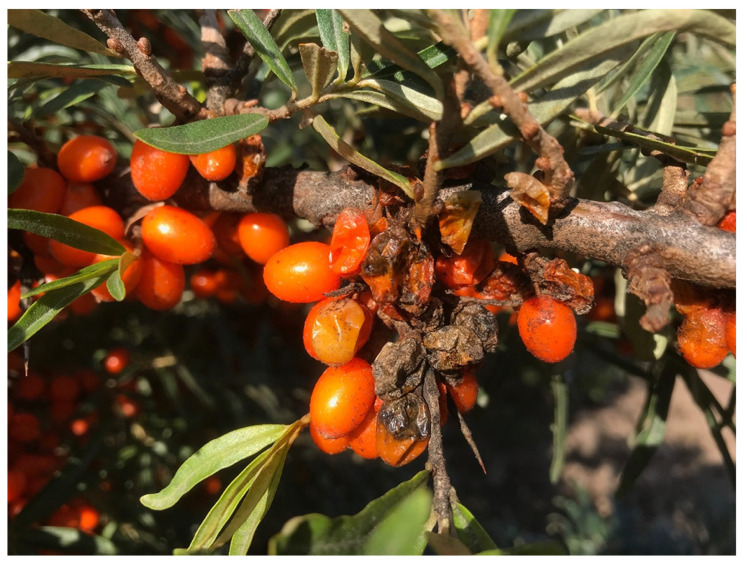
Damage caused by sea buckthorn fruit fly *Rhagoletis batava*.

Numerous insects have been reported to infest sea buckthorn orchards around the world [3]. In China, the most dangerous pest is *Holcocerus hippophaecolus*, found in plantations in, e.g., the Liaoning province and Inner Mongolia, where more than 0.8 million ha have been damaged. As of yet, there is no information on cultivar-dependent variation in susceptibility to this pest. Outside of China, the sea buckthorn fruit fly *(Rhagoletis batava)* is potentially the single biggest threat to commercial production. Infestations can decimate fruit yield and ultimately lead to total loss of the harvest (Figure 8). This fly probably originates from Siberia and has gradually spread west from Siberia into Eastern Europe and south towards Mongolia and China. Severe damage has been reported in commercial orchards in the Baltic countries, Scandinavia, Poland, Romania, and Germany, with yearly yield losses. In China, the extent of fly damage is currently less prevalent, possibly because this insect has not yet adapted to the climate.

Susceptibility to the sea buckthorn fruit fly has been reported to vary among cultivars, with ‘Baikal’ being highly resistant [62]. Early-ripening cultivars may be more susceptible since their fruits are at a suitable stage for egg-laying. Fruit size, color, and shape may also play a role since female flies of this genus are known to evaluate these traits carefully before laying their eggs. Thus, the majority of relatively resistant varieties appear to have small, reddish, and late-ripening fruit [98]. Proper studies of the inheritance for this trait have, however, not yet been presented.

Molecular mechanisms involved in insect resistance are not yet well documented, but brassinosteroid (BR) appears to be involved in the stress tolerance of many plant species. In sea buckthorn, BR content was much higher in fruits infected by the sea buckthorn fruit fly compared to uninfected fruits [99]. Moreover, since damage was lower in fruit treated with BR, this compound may enhance resistance in sea buckthorn. Several BR biosynthesis-related *HrCYPs* genes were identified in a transcriptome analysis. The most up-regulated gene in infected sea buckthorn fruits was *HrCYP90B1*, which may act as a positive regulator in resistance to the sea buckthorn fruit fly.

### 7.5. Chemical Contents

An increasing part of the sea buckthorn cultivation is aimed at producing raw materials for the health food, medicinal, and cosmetic industries. Research has therefore focused on medicinal properties in mainly fruits, with special emphasis on vitamins, phenols, lipids, phytosterols, carotenoids, tocopherols, and triterpenoids. Sea buckthorn is indeed rich in many bioactive phytochemicals, but the contents of the fruit pulp, seeds, and leaves vary considerably. This variation can be attributed to species, ripening time, cultivar, harvest date, influence of year, as well as their interactions, as noted for, e.g., content of acids, sugars and sugar alcohols [100], carotenoids [101], tocopherols [102]), and total phenolic compounds [103]. Also, location [104], orchard management, and sample treatment can influence the reported results, and therefore published data are not easily compared. Future analyses of chemical compounds in fruits should be conducted on samples where the maturity stage is more precisely defined using, e.g., pheophytin as a maturity marker [101].

Ideally, phenotyping data are obtained from samples of replicated plants in formal multi-year field trials, but most sea buckthorn studies are instead based on a limited number of samples without true biological replicates, and only a few studies have focused on the comparison of several species and subspecies. Furthermore, a lack of cheap and efficient biochemical analytical methods has historically restricted the study of phytochemical variation, but several advanced Liquid Chromatography–Mass Spectrometry (LC-MS) and High-Performance Liquid Chromatography–Mass Spectrometry (HPLC-MS) methods are now commonly used to provide reliable data. Fast and efficient rapid proton Nuclear Magnetic Resonance spectroscopy (^1^H NMR) analysis has been used for the metabolic profiling and discrimination of wild sea buckthorn fruits grown at different locations in Finland (subsp. *rhamnoides*) and China (subsp. *sinensis*) [104]. The two subspecies and different growth sites could be distinguished, and variation within subsp. *rhamnoides* was shown to be associated mainly with the higher temperature, solar radiation, and humidity, as well as the lower precipitation in southern Finland, yielding higher levels of O-ethyl β-d-glucopyranoside and d-glucose and lower levels of malic, quinic, and ascorbic acids. Significant metabolic differences in genetically identical fruits were observed between latitudes 60° and 67° north in Finland. High altitudes (>2000 m) were correlated with higher levels of malic and ascorbic acids in subsp. *sinensis*.

Using NMR, effects of location were also studied on samples of subsp. *rhamnoides* cultivars grown in northern and southern Finland as well as in Canada, showing that the metabolic profile of the northernmost fruits was distinctly different from those grown in southern Finland or Canada, thus demonstrating considerable plasticity in the acclimatization to growth environments [105]. In another study, NMR metabolomics and multivariate data analysis were used to study variation in seven species and seven subspecies of *Hippophae* (90 accessions), with metabolites being quantified with quantitative NMR (qNMR) [106]. Different species were clearly discriminated against by their content of organic acids, L-quebrachitol, and carbohydrates.

NMR non-targeted metabolomics have also been applied to the leaves of sea buckthorn. In a study of two cultivars (subsp. *rhamnoides*) grown in the south and north of Finland during two consecutive growth seasons, the highest variance in the metabolic profile was linked to the growth stage, with the second highest variance attributed to location [107]. The north–south comparison identified fatty acids and sugars as discriminatory metabolites.

An NMR-based approach was used on sea buckthorn juice to reveal metabolic changes during fermentation with different strains of *Lactiplantibacillus plantarum* [108]. In total, 46 metabolites were identified from the fresh and fermented juice, including various sugars, amino acids, organic acids, ketones, nucleosides, and one alkaloid. Thus, NMR-based metabolomics could be a useful approach for simultaneous metabolic profiling, species and subspecies differentiation, and quality assessment of sea buckthorn, as well as for discriminating the effects of the geographical origin of sea buckthorn fruits and leaves.

Sugars and sugar alcohols: Fructose, glucose, and ethyl glucose are usually the most abundant sugars in sea buckthorn fruits, and L-quebrachitol is the most abundant sugar alcohol, with methyl-myo-inositol and myo-inositol being present in smaller amounts. Interestingly, changes in sugar content during maturation seem to differ between species. Levels of fructose and glucose in subsp. *sinensis* fruit juice increased during ripening, whereas corresponding contents in samples of subsp. *rhamnoides* decreased [100].

Fruits were collected from nine natural stands of subsp. *sinensis* in China during three years, and the influence of latitude and altitude on sugars and sugar alcohols was investigated [109]. Although samples were collected at optimal maturity, the contents of fructose, glucose, and L-quebrachitol in the fruit juice varied widely: 0.01–7.17, 0.05–7.85, and 0.21–1.09 g/100 mL, respectively. The contents of fructose, glucose, and total sugar correlated positively with the growth latitude but negatively with the altitude. The contents of L-quebrachitol correlated strongly and positively with latitude.

In a study of inositols and methylinositols in the juice of three subspecies, wild Chinese fruits (subsp. *sinensis*) contained higher levels of L-quebrachitol (average 615 mg/100 mL juice) and methyl-myo-inositol (average 58 mg/100 mL juice) than the Finnish (subsp. *rhamnoides*, 276 and 11 mg/100 mL juice, respectively) and Russian (subsp. *mongolica*, 228 and 16 mg/100 mL juice, respectively) fruits [110]. The content of myo-inositol was higher in the Chinese and the Russian fruits than in the Finnish (26 and 20 mg/100 mL juice vs. 8 mg/100 mL juice). In the Chinese fruits, the contents of methyl-myo-inositol and L-quebrachitol increased, whereas that of myo-inositol decreased from late September to late November. The content of L-quebrachitol in the Chinese fruits correlated negatively with the air temperature and the number of frost-free days.

In another study, sugars, ethyl β-d-glucopyranose, and methylinositol were analyzed in fruit juice from three subspecies (subsp. *sinensis*, *rhamnoides*, and *mongolica*) collected in China, Finland, and Russia during four years [100]. Origin and harvesting date had a significant impact on the content of sugars. During the harvesting period, sugar content developed differently in fruits of the different subspecies. Fructose (subsp. *sinensis* 1.5–11.7 g/100 mL, subsp. *rhamnoides* 0.1–0.6 g/100 mL, and subsp. *mongolica* 0.9–4.3 g/100 mL) and glucose (subsp. *sinensis* 1.6–12.5 g/100 mL, subsp. *rhamnoides* 0.8–2.9 g/100 mL, and subsp. *mongolica* 4.3–7.2 g/100 mL) were the main sugars in all three subspecies. Ethyl glucose was present in the sugar fraction of subsp. *rhamnoides* (0.1–1.9 g/100 mL) but was found only in trace amounts in the other two subspecies. In subsp. *rhamnoides*, the level of ethyl glucose increased during the harvesting period and was accompanied by a decrease in glucose content. Methylinositol was present in higher levels in subsp. *sinensis* (0.3–1.6 g/100 mL) than in the other two subspecies (0.1–0.5 and 0.2–0.3 g/100 mL for subsp. *rhamnoides* and subsp. *mongolica*, respectively). The total amount of sugars was highest in subsp. *sinensis* and lowest in subsp. *rhamnoides*.

Organic acids and vitamin C: Sea buckthorn is a rich source of organic acids, mainly malic, quinic, and ascorbic acids. Maturity has a significant influence on the content of ascorbic acid, with an average reduction of 25% from early to late maturity reported for, e.g., hybrid cultivars derived from subsp. *mongolica* [111] as well as for wild-growing subsp. *rhamnoides* [112]. The time of sampling is therefore crucial to obtaining comparable data. Ascorbic acid seems to be especially high in the fruits of *H. salicifolia*, but also *H. rhamnoides* subsp. *sinensis* and subsp. *yunnanensis* have a high content [113].

Wild fruits of subsp. *sinensis* are reported to contain 5−10 times more vitamin C in the juice fraction than fruits of subsp. *rhamnoides* and subsp. *mongolica* [112]. In subsp. *sinensis*, the content of organic acids in fruits has been shown to vary among genotypes and sites [109]. Malic acid (1.55–8.84 g/100 mL juice) and ascorbic acid (0.25–1.66 g/100 mL) thus increased as the altitude increased and as the latitude decreased, while the content of quinic acid (0.07–2.94 g/100 mL) correlated strongly and positively with the latitude.

In another study, organic acids were analyzed in the fruit juice of three subspecies over four years [100]. Similarly, as for sugars, growth location and harvesting date had a significant impact on the content of acids. The content of malic acid decreased from initial ripening to full ripening and thereafter increased slightly. Malic acid content varied in all three subspecies (subsp. *sinensis* 1.9–9.2 g/100 mL, subsp. *rhamnoides* 2.3–4.7 g/100 mL, and subsp. *mongolica* 0.8–2.7 g/100 mL), as did quinic acid (subsp. *sinensis* (0.7–7.5 g/100 mL, subsp. *rhamnoides* 0.7–4.3 g/100 mL, and subsp. *mongolica* 1.3–2.6 g/100 mL).

Tocopherols, tocotrienols, and vitamin E: Both tocopherols and tocotrienols have vitamin E activity, with α-tocopherol being the most important in the fruit flesh (pulp) of sea buckthorn. In seed oil, the content of vitamin E reported for different sea buckthorn species (*H. goniocarpa*, *H. gyantsensis*, *H. neurocarpa*, *H. rhamnoides*, *H. salicifolia*, and *H. tibetana*) and subspecies was in the range of 98–273 mg per 100 g of oil, with the highest amount for *H. neurocarpa* subsp. *stellatopilosa* and the lowest for *H. tibetana* [113]. In the same plant material, vitamin E content in pulp oil ranged from 54 to 181 mg per 100 g of oil, with the highest amount for *H. goniocarpa* subsp. *goniocarpa* and the lowest for *H. neurocarpa* subsp. *stellatopilosa* [113].

Seeds of subsp. *sinensis* contained fewer tocopherols and tocotrienols (average 130 mg/kg) compared with seeds of subsp. *rhamnoides* (average 290 mg/kg) and *mongolica* (average 250 mg/kg) in a large study with both wild and cultivated material [112]. By contrast, the fruit flesh of subsp. *sinensis* berries had 2−3 times higher contents of tocopherols and tocotrienols compared to the other two subspecies (120 mg/kg vs. 40 mg/kg in subsp. *rhamnoides* and 50 mg/kg in subsp. *mongolica*). Overall, the fresh whole fruits of subsp. *sinensis* were clearly the best source of total tocopherols and tocotrienols. In the same study, it was shown that the total content of tocopherols and tocotrienols in fruit flesh reached its maximal level around early to mid-September, whereas the content in seeds continued to increase until the end of November.

In a study of four cultivars over three years, ’Ljubitelskaya’ (synonym ‘Botanicheskaya Ljubitelskaya’), derived from a cross between subsp. *mongolica* and subsp. *rhamnoides*, generally had a higher content of most tocopherols and tocotrienols in the fruit flesh than the other cultivars studied [102]. The total amounts of tocopherols plus tocotrienols were two-fold higher compared with ‘Eir’. The most extreme difference between the cultivars was noted for δ-tocopherol, with the highest amounts in ’Ljubitelskaya’ and only trace amounts in ‘Eir’. Levels of α-tocopherol were higher early in the ripening period, while at later dates, δ-tocopherol levels increased.

Carotenoids and vitamin A: In the fruit flesh of sea buckthorn, β-carotene, γ-carotene, and lycopene are the most important carotenes, while zeaxanthin, lutein, and β-cryptoxanthin are the most important xanthophylls. Vitamin A activity stems mainly from the content of β-carotene. In *H. rhamnoides*, β-carotene constitutes approximately 15–55% of the total carotenoids, with a range of 100–500 and 20–100 mg/100 g in pulp and seed oil, respectively [114].

The fruit pulp of four cultivars was analyzed for carotenoid contents during ripening in three consecutive years [101]. The different carotenoids generally increased in concentration during ripening and comprised from 120 to 1425 μg/g dw (dry weight) of total carotenoids (1.5–18.5 mg/100 g of pulp fresh weight) depending on cultivar, harvest time, and year. The analyses showed that the effect of cultivar was considerably larger than the effect of year and harvest time.

Lipids and fatty acids: Sea buckthorn fruits are rich in oils, both in pulp and seeds. The oil content is generally lower in the pulp compared to the seeds when based on pulp fresh weight, but larger when based on dry weight. In a study of 78 accessions, oil content proved to be higher in pulp (3.5–38.6% dw) than in seeds (3.9–12.8% dw), especially in subsp. *mongolica* accessions [58]. The lowest total oil content in *H. rhamnoides* has been reported for subsp. *sinensis* (2–3%, whole fruit fresh weight), whereas subsp. *turkestanica* and subsp. *mongolica* have the highest (4–14% and 2–10%, respectively) [114]. In *H. goniocarpa*, *H. gyantsensis*, *H. rhamnoides* (including all subspecies except subsp. *caucasica*, for which data are missing), *H. salicifolia*, and *H. tibetana*, pulp oil content ranges between 1.6 and 7.8% (fresh weight), but is considerably higher in *H. neurocarpa*, 10.6–19.8% [113]. Seed oil content ranges between 5.5 and 13.0% in *H. goniocarpa*, *H. gyantsensis*, *H. rhamnoides*, and *H. salicifolia* but is higher in *H. neurocarpa* and *H. tibetana* (8.6–16.2% and 15.3–19.6%, respectively).

The fatty acid profile differs between pulp oil and seed oil, but the major fatty acids are apparently the same in the commonly cultivated subspecies, i.e., subsp. *caucasica*, subsp. *mongolica*, subsp. *rhamnoides*, and subsp. *sinensis*. The content of monounsaturated fatty acids is over 50% in the pulp oil of subsp. *mongolica*, subsp. *rhamnoides*, and subsp. *sinensis* (51–59%), but lower for subsp. *caucasica* (27–43%) [58,89]. The content of polyunsaturated fatty acids is 10–20% in subsp. *mongolica*, subsp. *rhamnoides*, and subsp. *sinensis*, but higher for subsp. *caucasica* (28–43%) [89]. Total content of polyunsaturated fatty acids is much higher in seed oil (55–78%) compared to in pulp oil (27–59%), but variation among subspecies of *H. rhamnoides* seems to be as large as or larger than the variation among species when many accessions are investigated [58].

The development of cultivars with high total oil content and high content of the rare palmitoleic acid, which can increase basal and insulin-stimulated glucose uptake and reduce de novo fatty acid synthesis and activity of lipogenic enzymes, should be possible using the genetic variation within and among species and subspecies. Recently, the main enzymes involved in pulp oil accumulation in sea buckthorn were identified [6,115]. A Real-Time Quantitative Reverse Transcription-Polymerase Chain Reaction (qRT-PCR) analysis of 15 genes involved in fatty acid and triacylglycerol (TAG) biosynthesis in two sea buckthorn genotypes helped to understand the mechanism of high C16:1n7 accumulation in fruit pulp [116]. A thorough understanding of the lipid synthesis pathway and its mechanisms for regulation is important and could in the future facilitate breeding of sea buckthorn cultivars with high oil contents and a specific composition of fatty acids using gene editing tools.

Phenolic compounds: Phenolic compounds are a very large group of phytochemicals present in all parts of the sea buckthorn plant, with the major phenols being flavonols (quercetin, isorhamnetin, and kaempferol derivatives), polymeric procyanidins, flavan-3-ols, and phenolic acids. In a study of cultivars derived from subsp. *mongolica* grown in Poland, leaves had the highest content of flavonols (921.7 mg/100 g dw) and branches the lowest (41.3 mg/100 g dw), with skin, flesh, seeds, and endocarp in between [117] (Table 2). Leaves also had the highest content of phenolic acids, albeit still low. The highest content of flavan-3-ols and polymeric procyanidins was found in branches and seeds, followed by leaves. The procyanidins in sea buckthorn are of the B-type [118]. Among flavonols, leaves are especially rich in quercetin derivatives. Quercetin derivatives were also high in skin and fruit flesh, followed by isorhamnetin derivatives and kaempferol derivatives [117].

Flavonol glycosides were identified and quantified in the fruits of plants grown in Finland and Canada but derived from wild-growing subsp. *sinensis* in China and cultivated subsp. *mongolica* [119]. Twenty-six flavonol glycosides were found, with isorhamnetin and quercetin as the major aglycones. The contents of flavonol glycosides ranged from 23 to 250 mg/100 g of fresh fruits and were significantly higher in subsp. *sinensis* than in subsp. *mongolica*. Among the subsp. *mongolica* cultivars, the fruits of ‘Oranzhevaya’ had the lowest (23 mg/100 g) content, and those of ‘Prevoshodnaya’ had the highest (80 mg/100 g) flavonol glycosides. Also, samples grown in Kittilä (northern Finland) had higher levels of most flavonol glycosides than samples grown in Turku (southern Finland) and Québec. For subsp. *sinensis*, the contents of most compounds increased as the altitude of the growth site increased and as the latitude decreased. Fruits from stands originally collected in Sichuan had remarkably high contents and unique profiles of flavonol glycosides.

Differences were found between subsp. *rhamnoides* and subsp. *mongolica* accessions, as well as for procyanidins [118]. Furthermore, samples of subsp. *rhamnoides* grown in northern Finland were characterized by a high amount of total procyanidins, typically 2–3 times higher than found in samples grown in Southern Finland. In subsp. *sinensis*, altitude did not correlate with the PA (proanthocyanidins) composition [118].

The flavonol profile of sea buckthorn fruits has turned out to be useful in separating species, and a fingerprint method based on 12 flavonoids identified from HPLC chromatograms has been developed [120]. Potentially, this method could also be used for quality assurance of sea buckthorn fruits and extracts.

In a recent study of two subsp. *mongolica* accessions with similar flavonoid profiles, chalcone synthase (CHS) and flavanone-3-hydroxylase (F3H) were the main enzymes responsible for the difference in flavonoid synthesis and accumulation in sea buckthorn fruits [6], which is in agreement with previous studies [121]. A deeper understanding of the flavonoid synthesis pathway could help breeders develop strategies for future breeding.

The content of different phenolic compounds is strongly influenced by developmental stage, as shown for both leaves [122] and fruits [6]. A proper sampling strategy must therefore be applied to obtain reliable and comparable data.

Sterols: Phytosterols can lower serum cholesterol levels in humans and therefore constitute an important group of bioactive compounds in plants. In a study of pulp oil in eight subsp. *mongolica* cultivars, 14 phytosterols were identified and quantified, the major compounds being β-sitosterol, 24-methylenecykloartanol, and squalene [123]. Although β-sitosterol was always the major sterol, the total amount and profile differed widely among cultivars (Table 3). In another study, sterols were analyzed in seeds, pulp/peel fractions, and whole fruit samples derived from two major subspecies (subsp. *sinensis* and subsp. *rhamnoides*) from Finland and China [124]. Total sterol contents in the seeds, the fresh pulp/peel, and the whole fruits were 1200–1800, 240–400, and 340–520 mg/kg, respectively. The corresponding values in the extracted oils were 12–23, 10–29, and 13–33 g/kg. β-sitosterol constituted 57–76 and 61–83%, respectively, of the seed and pulp/peel sterols. The sterol content and composition showed little variation between subspecies and collection sites. Different harvesting dates showed significant effects on the levels of some sterols, both in the seeds and in the pulp/peel. The content of sterols can depend on the extraction method, and supercritical carbon dioxide has proven to be an efficient and reliable method for exhaustive extraction of sterols [125].

Triterpenoids: The pharmaceutical potential makes triterpenoids a relevant target for future breeding efforts. Recently, a detailed account of triterpenoids in the skin, flesh, endocarp, seed, leaves, and branches of cultivars derived from subsp. *mongolica* was given [117] (Table 4). The 11 identified triterpenoids were divided into two groups according to the amount detected: (1) betulin, betulinic acid, corosolic acid, maslinic acid, pomolic acid, oleanolic acid, and ursolic acid, all of which were present in significant amounts, and (2) α-boswellic acid, erythrodiol (only in branches and leaves), tormentic acid, and uvaol, all found in amounts below 1 mg/100 g dw [117]. In fruit fractions (skins, flesh, endocarp, and seed), pomolic acid dominated and constituted 62% of total triterpenoids in the fruit flesh. The highest content of maslinic acid was also found in the fruit flesh, constituting 16% of total triterpenoids. The highest content of corosolic acid and betulinic acid was found in branches. Skins had the highest amounts of oleanolic acid, ursolic acid, and betulin. Leaves had similar levels of ursolic acid as skins; in leaves, it constituted 42% of total triterpenoids. Large differences were also found among cultivars; thus, ‘Botanicheskaya Ljubitelskaya’ had the highest amounts of triterpenoids in skins and seeds, whereas ‘Golden Rain’ and ‘Tatiana’ had the highest triterpenoid levels in branches and the lowest in seeds. Thus, breeding strategies should consider both genotype and utilized plant parts in order to optimize the contents.

Sensory aspects: In many countries, sea buckthorn juice is the main or only product derived from commercial plantations. Naturally, the amount and balance of chemical compounds that affect sensory components are crucial for cultivars grown mainly for their culinary properties. Astringency, sourness, and bitterness were negatively correlated with pleasantness and favored only by a few members of a sensory panel in Finland testing sea buckthorn juice, whereas sweetness showed a strong positive correlation with pleasantness [126]. In that study, genotypes obtained from crosses between subsp. *rhamnoides* and subsp. *mongolica* gave the most acceptable juice, possibly due to a lower total acidity. In another Finnish study, the total content of sugars and the sugar/acid ratio correlated positively with sweetness and negatively with sourness and astringency in the juice of six cultivars derived from subsp. *mongolica* and one cultivar from subsp. *rhamnoides* [127]. Total acidity and titratable acidity instead correlated positively with sourness and astringency and negatively with sweetness.

The slightly astringent and bitter taste of sea buckthorn fruits has also been shown to correlate with the contents of flavonols, proanthocyanidins, and ethyl b-D-glucopyranoside [128,129]. However, the low average degree of procyanidin polymerization (number of flavan-3-ol units in polymers) ranging from 2.4 (for branches), followed by 2.6 (leaves), 4.4 (fruit flesh), to 8.0 (for seeds), and the presence of preferably dimeric and oligomeric flavan-3-ols indicate a potentially low intensity of astringency in food applications [117].

Besides these non-volatile compounds, odor-active volatiles have a crucial influence on the sensory quality of sea buckthorn fruits [130]. Fatty acid oxidation during the late ripening stages results in unattractive fragrances and off-flavors in many cultivars, but breeding and selection can help reduce rancidity, as shown in several of the recently developed cultivars in Sweden.

Fruit color: Sea buckthorn fruit color is an important commercial trait and varies across species from bright yellow, over orange and red, to brown in mature fruits. Integrative analyses of the transcriptome and targeted metabolome, including carotenoids, flavonoids, and chlorophylls, were recently performed for five accessions: subsp. *sinensis* ‘FengNing’ (yellow), subsp. *mongolica* ‘XiangYang’ (orange), and two accessions of red-colored and yellow-colored hybrid offspring, as well as a brown *H. neurocarpa* subsp. *neurocarpa* fruit [131]. A total of 209 flavonoids and 41 carotenoids were identified, but a high content of chlorophyll was found only in the brown fruits. It was shown that the quantities and relative proportions of the flavonoids, carotenoids, and chlorophyll led to the different colors of the sea buckthorn fruits, and key genes related to the carotenoids and chlorophyll metabolism were identified. The high content of chlorophyll in the brown fruit was closely related to the downregulated expression of key genes in the chlorophyll degradation pathway. In yellow fruits, carotenes dominated, while lycopene contributed significantly to the color in orange and red fruits. Future breeding efforts could therefore target key enzymes affecting the synthesis and accumulation of carotenoids and chlorophylls.

## 8. Breeding of Male Plants

Male sea buckthorn plants are not given as much attention from breeders as the females since their only role is usually to provide prolific amounts of pollen. Male plants are therefore selected mainly for plant vigor, persistence, and the timing of pollen dispersal, which should overlap with the flowering time of the female plants. Insufficient cold hardiness has, however, been reported as a major problem with continental male cultivars, making them unsuitable for production in Finland and the Baltic countries [67]. Another potential goal is to produce plants for commercial pollen-based medicinal products [132]. Since sea buckthorn is a dioecious wind-pollinated species, the inheritance of fruit characteristics from male plants can only be evaluated from the performance of their offspring following controlled crosses.

Two thornless male cultivars with winter-hardy flower buds were selected at the Lisavenko Research Institute in Russia [59]. ’Aley’ is very vigorous and is mainly recommended for commercial plantations, whereas the compact ’Gnom’ is suitable both for commercial plantings and home gardens. In China, ‘Wucixiong’ was selected from wild stands of subsp. *sinensis*, whereas the almost thornless ‘Mengzhongxiong’ derives from subsp. *mongolica* × subsp. *sinensis.*

**Figure 9:**
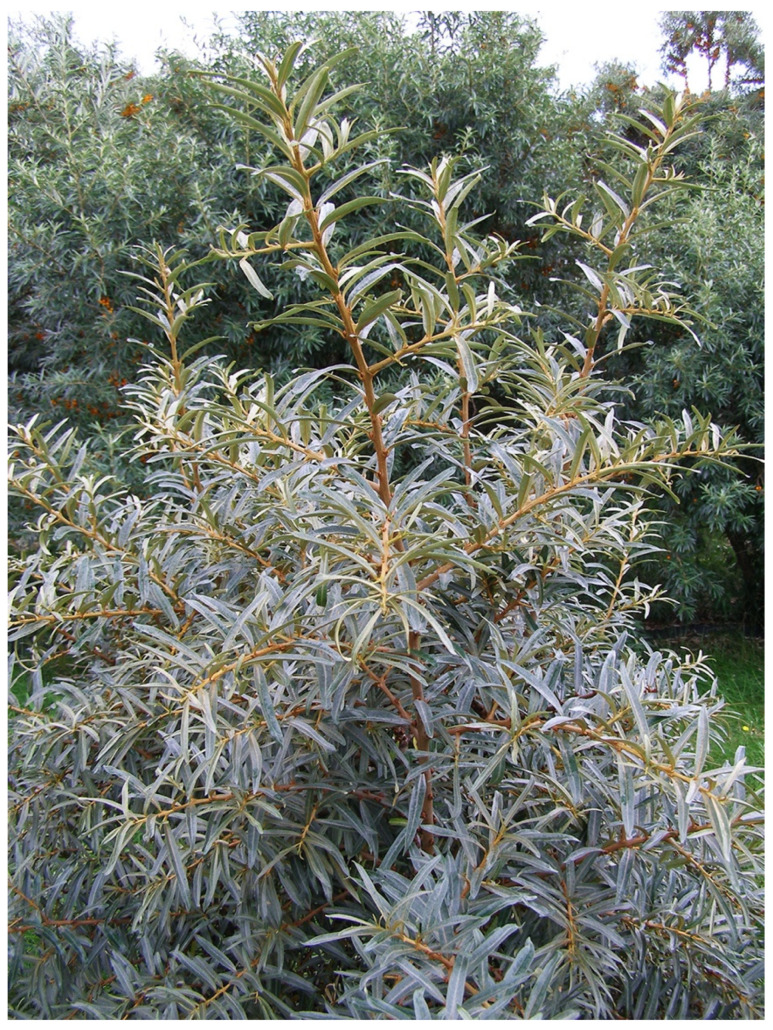
‘Svenne’, male Swedish cultivar (*Hippophae rhamnoides* subsp. *rhamnoides*).

’Pollmix’ is the common name for a group of four different genotypes selected from wild stands of subsp. *rhamnoides* in Germany [60], while ‘Tarmo’ was selected from wild stands of subsp. *rhamnoides* in Finland, and ‘Edgars’ and ‘Skibes vir’ in Latvia. Later on, ‘Lord’ was selected in Latvia from a cross between a subsp. *mongolica* × subsp. *rhamnoides* hybrid and local subsp. *rhamnoides.* In Sweden, ‘Romeo’ was selected from a subsp. *mongolica* × subsp. *rhamnoides* population, and ‘Svenne’ was selected from an open-pollinated local subsp. *rhamnoides* selection grown in a breeding collection (Figure 9). More recently, ‘Balsgård Hubert’ was released, a vigorous male selected from an open-pollinated local selection of a hybrid between subsp. *mongolica* and subsp. *rhamnoides*.

Although sea buckthorn is a predominantly dioecious plant, hermaphrodite flowers sometimes develop on normal male plants [64]. Thus, a future breeding goal could be to develop truly hermaphrodite plants, which could potentially exclude the need for male pollinator plants.

## 9. Breeding Methods

### 9.1. Conventional Breeding Methods

Sea buckthorn breeding started with the mass selection of superior plants in wild populations. Mass selection is still used to ensure locally adapted plant material for use in breeding programs. However, hybridization has increasingly replaced mass selection as a breeding method.

For hybridization, flowers need to be isolated 2–3 days prior to the beginning of flowering, preferably using bags of dense fabric to cover branches on female plants. A few days later, branches with open male flowers are placed together with the isolated female flowers, and bags are shaken the following day to ensure good pollination. A different approach consists of removing the bags (which requires very calm weather with absolutely no wind) and carefully dusting the female flowers with previously collected pollen. The female flower can receive pollen for about a week, depending on the temperature. The bags must, however, be removed when the stigma is no longer receptive to allow proper ovary development, which is dependent on light [133].

Sea buckthorn pollen can be collected from forced twigs being cut 2–3 days before flowering. The pollen grains can be stored cool and dry for later use, but little information is available on the possibility of long-term storage of pollen and its viability. Germination of pollen grains is reported to occur in 3–4 h and fertilization takes 5–10 days, depending on temperature [134].

Another approach is to grow female and male plants in pots and place them together in a greenhouse chamber for hybridization. It is important to shake the male plants daily to ensure proper pollen dispersal and pollination.

As soon as the fruits have ripened, seeds can be collected, stored dry, or immediately sawn since they have a short-term physiological dormancy [135]. However, cold stratification for 15–90 days reduces dormancy and improves germination of *H. rhamnoides*. Optionally, soaking seeds in water for 7 days could replace stratification [136]. Seeds can maintain germinability for more than two years when stored at low humidity, even at room temperature [135].

Sea buckthorn seedlings enter the adult stage after 3–5 years and can then be evaluated for fruit characteristics and yield potential. Selected seedlings are propagated for comparative field trials. Sea buckthorn is easily propagated by hardwood and softwood cuttings [137], but it can also be micro-propagated [138]. Following propagation, plants usually start to yield after 3-4 years and should be evaluated for 3 years at least in observation trials before superior selections are propagated for large-scale yield trials in different locations. The time taken from initial cross to release of cultivars is thus at least 15 years.

### 9.2. Treatment with Mutagenics

Induced mutagenesis with a sub-lethal dose of ionizing irradiation or with chemicals can increase the genetic variability of sea buckthorn and shorten the time required for developing a new variety [139]. Using irradiation, several cultivars have been developed in Russia, e.g., ‘Druzhina’, ‘Podrugha’, ‘Zolotoy Kaskad’, ‘Ivushcka’, ‘Ognistaya’, ‘Zarnitsa’, and ‘Krasny Fakel’. Some of these are reported to have large aromatic fruits, a high yield, a high content of vitamins and other biologically active substances, long pedicels, and a low number of thorns [64]. ‘Elizaveta’, ‘Inya’, and ‘Sudaruschka’ were instead derived as chemically induced mutants of ‘Panteleevskaya’ and are presently being marketed for qualities like high yield, high carotenoid contents, and a short juvenile period.

Polyploid sea buckthorn plants (2n = 24) can be found in wild populations but have also been developed using colchicine [140]. Although polyploidization frequently results in abnormal and weak plants, it could also contribute to novel and useful variation.

### 9.3. Molecular Marker-Assisted Breeding

Although considerable initial progress can be attained from choosing parents with above-average performance in desirable traits, DNA marker-based selection can be a valuable tool, especially for traits that are difficult to evaluate in the field. DNA markers are now being used to screen putative parents in many fruit and berry crops for traits like disease resistance and fruit texture components. The cost-effectiveness of screening the resulting seedlings depends on the percentage of seedlings that can be discarded at an early stage, which in turn depends on the number and efficiency of the DNA markers scored, as shown for apples [141].

In sea buckthorn, very few molecular markers have been developed as of yet, apart from those used for sex determination. Some attempts for disease resistance have been made. Using a disease index for resistance/susceptibility to DSD, four ISSR markers [142] and eleven Sequence-Related Amplified Polymorphism (SRAP) markers were shown to be associated with the number of disease symptoms in the investigated plant material [143]. These multilocus marker methods do, however, not lend themselves well to large-scale screenings. Also, the identity of the fungi causing DSD varies considerably between locations as well as the type of germplasm. For the identification of causative genes and the development of single-locus DNA-based markers, large amounts of phenotypic data must first be collected, preferably based on carefully conducted inoculation experiments with properly identified fungal strains. In the case of sea buckthorn, this will first require considerable research into the plant pathology of the species.

The detection of key genes for important traits in other crops has been attempted via different avenues. Traditionally, major genes and Quantitative Trait Loci (QTL) are identified based on a molecular-marker mapping population with offspring that segregate for the trait of interest. So far, really dense (marker-rich) genetic maps have, however, not been published for sea buckthorn. Another, more recent, and perhaps more promising approach is to conduct a GWAS, which exploits the linkage disequilibrium present among individuals from natural populations or germplasm collections. These are usually more diverse than segregating progenies and can be used to map QTL with high resolution. GWAS is facilitated in many crops by the recent development of high-density SNP arrays with uniform coverage of the whole genome. Genes can, however, also be located using the increasingly affordable methods for whole-genome re-sequencing (see below).

The identification of important genes and the development of molecular markers for use in genetic research and plant breeding are facilitated by the existence of a chromosome-level genome assembly obtained via in-depth sequencing. This has been lacking for the entire family Elaeagnaceae, but no less than four genomes were published in 2022, three of which were developed for *Hippophae* [140,144,145].

The first of these was *H. rhamnoides* subsp. *mongolica* cultivar ‘Sunny’, with a total genome length of 849.04 Mb [144]. The quality and completeness of this assembly were ascertained, and high synteny between the physical map and the genetic map was also demonstrated. A total of almost 31,000 genes were predicted with an average length of 4900 bp and an average coding-sequence length of 1307 bp. Two sequential whole-genome duplication events were proposed, the first occurring about 36–41 million years ago (Mya) and the second 24–27 Mya. These events have prompted an expansion of genes related to ascorbate and aldarate metabolism, lipid biosynthesis, and fatty acid elongation. Several key genes contributing to the high contents of polyunsaturated fatty acids and ascorbic acid were identified via transcriptome sequencing. A subsequent population genomic analysis based on whole-genome sequencing of 55 *H. rhamnoides* accessions (wild and cultivated subsp. *mongolica* and wild subsp. *sinensis*) yielded a large number of SNPs that were used for analysis of genetic relatedness. A search for candidate genomic regions under positive selection indicated that selective sweeps have contributed to the richness of fatty acids and ascorbic acid in the fruits of domesticated subsp. *mongolica* plants [144]. Numerous genes for economically important traits were identified in the analyzed accessions, which will hopefully soon result in easy-to-use molecular tools for marker-assisted plant breeding.

The second genome assembly was undertaken with a sample of *H. rhamnoides* subsp. *sinensis* [145]. The obtained genome was 730.46 Mb, and many genes of importance for, e.g., vitamin C synthesis were identified. The sequenced genome was shown to share a high number of conserved symbiotic root nodulation genes with other species (e.g., *Lotus* and *Medicago*) that also have rhizobia-induced symbiosis.

The third genome assembly was developed for a different species in the *Hippophae* genus, namely *H. tibetana*, which is an extreme high-altitude plant growing at 3000–5200 m above sea level on the Qinghai–Tibet Plateau [40]. This genome had undergone two duplications, just like the genomes assembled for *H. rhamnoides* [144,145], but was somewhat larger, 1452.75 Mb, than the other two. An adaptation to the more extreme and variable conditions at high altitudes (cold, drought, extremely low supplies of nitrogen, low carbon dioxide concentration, and high UV-B ultraviolet light stress) is indicated by the high number of genes related to adaptation to high ultraviolet and low temperature in *H. tibetana*, as well as more copies of nitrogen-fixing genes.

In many crops, the development of molecular markers has been hampered by a lack of high-quality phenotyping data. In addition to exact and informative measurements, the potential to assess a genotype–environment interaction by measuring the same trait over several years—and preferably on replicated plants of the same genotype growing in different orchards—would be very valuable. In apple, large field collections have recently been implemented for this purpose, such as the international REFPOP project with the same apple genotype being planted at six different locations [146]. A similar approach in sea buckthorn could be very useful, but except for the joint Russian–Chinese project “Transcriptional regulation mechanisms of tissue-specific expression of key genes involved in lipid biosynthesis in sea buckthorn” (2023–2025), large international co-operative projects have been few for this crop.

## 10. Summary and Outlook

DNA markers have been applied to quantify the amount of genetic variation between and within taxa and populations, as well as for discrimination among sea buckthorn accessions in germplasm collections. Modern analytical techniques have demonstrated that there is genetic variation in the biochemical compounds among different species, subspecies, and genotypes, but also an environmental impact from differences in harvesting time, storage conditions, and processing methods. Research involving transcriptomics, metabolomics, and proteomics has become very valuable for the identification of chemical pathways and the enzymes that are responsible for crucial steps.

Until now, there have been few studies on the inheritance of yield, chemical contents, resistance traits, or the identification of markers for desirable genes. Technical developments are, however, moving fast; large-scale genotyping-by-sequencing, together with improved data analyses such as GWAS and Genomics-Assisted Prediction (GAP), will most likely soon improve our understanding of the intricate networks that regulate economically important traits in sea buckthorn. Such information, together with the recently published reports on the sea buckthorn genome and improved phenotypic data, if these can be acquired, will be crucial in developing large DNA-based assays with carefully selected markers for key genes to be used in applied breeding programs. The need for high-quality phenotypic data must, however, be addressed, e.g., by large co-operative multi-site projects.

## Figures and Tables

**Table 1:** Female sea buckthorn cultivars of special interest in cultivation history and/or in plant breeding.

Cultivar	Interesting Trait(s)	Origin	Reference
Afina	Very vigorous, large fruits, long pedicels	Russia, subsp. *mongolica*	[59]
Altaiskaya	Thornless, sweet taste	Russia, subsp. *mongolica*	[59]
Askola	Dark orange fruit, high vitamin C and E contents	Germany, subsp. *rhamnoides*	[60]
Aurelia	High yield, large fruits, long pedicels	Russia, subsp. *mongolica*, offspring of ‘Avgustina’	[59]
Avgustina (Avgustinka)	Thornless, large fruits, long pedicels, early ripening	Russia, subsp. *mongolica*	[59,61]
Azhurnaya (Agurnaya)	Thornless, large fruits, long pedicels	Russia, subsp. *mongolica*	[59,61]
Baikal	Tolerant to sea buckthorn fly, sweet taste	Russia	[62]
Botanicheskaya Ljubitelskaya (Sunny)	High yield, large yellow fruits, low acidity, medium tolerant to sea buckthorn fruit fly	Russia, subsp. *mongolica* × subsp. *rhamnoides*	[62]
Chaoyang	Vigorous, late ripening, long and drooping branches	China, subsp. *mongolica* × subsp. *sinensis*	
Chechek	Thornless, compact stature, long period of maturity, high in carotenoids	Russia, subsp. *mongolica*	[59]
Chengse	Late ripening	Russia, subsp. *mongolica*	[61]
Chuyskaya (Star of Altai)	Thornless, compact stature, high yield, sweet taste, suitable for handpicking, early ripening	Russia, subsp. *mongolica*	[59,61]
Dalate	Thornless, balanced acidity/sweetness	China, subsp. *mongolica* × subsp. *sinensis*	[63]
Dar Katuni	Small fruits	Russia, subsp. *mongolica*	[59,61]
Desert maslichnyi (Maslichnaya)	Small reddish fruits, tolerant to wilt	Russia, subsp. *mongolica*	[59,62]
Dorana	Smallish stature, suitable for hand picking	Germany, subsp. *rhamnoides*	[60]
Druzhina	Compact, few branches, suitable for machine harvesting, large fruits with hard pericarps	Russia, subsp. *mongolica*, induced irradiation mutant	[64,65]
Eir	High yield, orange color, non-rancid	Sweden, subsp. *mongolica* × subsp. *rhamnoides* (BHi727102)	[9]
Elizaveta	High yield, large fruits, long pedicels, susceptible to *Fusarium* wilt	Russia, subsp. *mongolica*, induced chem. mutant of ‘Panteleevskaya’	[59,61]
Essel	High yield, large fruits, long pedicels, tolerant to *Fusarium* wilt, suited for fresh eating	Russia, subsp. *mongolica*	[59]
Etna	High yield, red fruits, very early ripening	Russia, offspring from open pollination of ‘Inya’	[59]
Eva	High sucrose content, suited for a cold climate with short summers, disease resistant	Latvia, subsp. *rhamnoides* × subsp. *mongolica*	
Ezhonghuang	Almost thornless, high acidity	China, subsp. *mongolica* × subsp. *sinensis*	[63]
Ezhongxian	Thornless, balanced acidity/sweetness	China, subsp. *mongolica* × subsp. *sinensis*	[63]
Fenja	Large fruits, red-orange color, low acidity, little flavor, easy harvest	Sweden, subsp. *mongolica* × subsp. *rhamnoides* (BHi72588)	[9]
Frugana	Vigorous, high yield, early ripening	Germany, subsp. *rhamnoides*	[60]
Gaoyou 1	Vigorous, high yield, high seed oil content	China, subsp. *mongolica* × subsp. *sinensis*	
Habego (Orange Energy)	Vigorous, high yield	Germany, subsp. *rhamnoides* × subsp. *mongolica*	
Harvest Moon	Early ripening, few thorns, winter-hardy, large fruits with long pedicels	Canada, subsp. *mongolica*	[66]
Hergo	Vigorous, high yield, late-ripening, suitable for machine harvesting, small fruits	Germany, subsp. *rhamnoides*	[67]
Hongji 1	Almost thornless, red rounded fruits	China, subsp. *mongolica* × subsp. *sinensis*	[68]
Hongxia	Ornamental, densely set orange fruits, fruits stay on branches for several months	China, subsp. *sinensis*	[69]
Hongyun	Tolerant to both low and high temperatures and to draught	Russia, subsp. *mongolica*	[61]
Hualin 1	Tolerant to both low and high temperatures and to draught	China, subsp. *mongolica* × subsp. *sinensis*	[63]
Hunjin	High yield, few thorns, large fruits, long pedicels	China, offspring from subsp. *mongolica*	[70,71]
Idun	Oblong orange fruits, non-rancid	Sweden, subsp. *mongolica* × subsp. *rhamnoides* (BHi727115)	
Inya	Arching branches, large red fruits with high carotenoid content, short juvenile period	Russia, subsp. *mongolica*, induced chem. mutant of ‘Panteleevskaya’	[59]
Ivushcka	Late ripening, red fruits with high carotenoid contents, can be harvested by shaking the bush	Russia, subsp. *mongolica*, induced irradiation mutant	[64,65]
Julia	Vigorous, high yield, orange fruits	Sweden, subsp. *mongolica* × subsp. *rhamnoides*	[9]
Kapriz (Caprice)	High sugar content	Russia, subsp. *mongolica*	[65]
Klavdia (Klavdija)	High yield, long pedicels, suitable for hand-picking, sweet taste	Russia, subsp. *mongolica*, offspring of ‘Chuyskaya’	[59]
Krasnoplodnaya	Large fruits, high carotenoid content, sensitive to wilt	Russia	[72]
Krasny Fakel	Late ripening, red fruits high carotenoid content, can be harvested by shaking the bush	Russia, subsp. *mongolica*, induced irradiation mutant	[64,65]
Leikora	Vigorous but with restricted root suckering, winter-hardy	Germany, subsp. *rhamnoides*	[60]
Lotta	High yield, winter-hardy, long pedicels, suited for hand-picking, low acidity, non-rancid	Sweden, subsp. *mongolica* × subsp. *rhamnoides* (Bhi 31415hona)	
Lvivyanka	High yield, large fruits	Ukraine, crosses with subsp. *mongolica*	[73]
Mariya	Low pull-out force (=fruits are easily detached)	Russia	[72]
Mary (Marija Bruvele)	Mild taste, high oil content, relatively tolerant to mycotic wilt and bud bacteriosis	Latvia, open pollination of ‘Botanicheskaya Ljubitelskaya’	[54]
Medova Osin	Drought tolerance, winter hardiness, disease resistance	Ukraine, subsp. *carpatica*	[73]
Mendeleevskaya	Tolerant to wilt	Russia	[62]
Mengzhonghong	Rounded fruits, balanced sweetness/acidity	China, subsp. *mongolica* × subsp. *sinensis*	[63]
Mengzhonghuang	Almost thornless, rounded fruits, balanced sweetness/acidity	China, subsp. *mongolica* × subsp. *sinensis*	[63]
Mukshanska	High yield, large fruits	Russia, offspring from open pollination of ‘Inya’	[73]
Nivelena	High yield	Russia	[72]
Novost Altaya	Small fruits, tolerant to Fusarium wilt	Russia, subsp. *mongolica*	[59,61]
Ognistaya	Late ripening, red fruits high in carotenoids, can be harvested by shaking the bush	Russia, subsp. *mongolica*, induced irradiation mutant	[64,65]
Ognivo	Thornless, large, and red fruits, long pedicels, late ripening	Russia, subsp. *mongolica*	[59]
Oranzhevaya (Orangenaya)	High yield, few thorns, small fruits	Russia, subsp. *mongolica*	[59,61,74]
Osinnia krasunia	High yield, large fruits	Ukraine, crosses with subsp. *mongolica*	[73]
Panteleevskaya	Large fruits	Russia, subsp. *mongolica*	[59]
Plamennaya	High yield, large fruits	Belarus	[61,72]
Podaruk Sadu	High yield	Russia	[72]
Podrugha	High sugar content	Russia, subsp. *mongolica*, induced irradiation mutant	[64,65]
Qiuyang	Drought tolerant	China, open pollination of subsp. *mongolica* ‘Wulangmu’	[75]
Raisa	Disease resistant, high acidity	Finland, subsp. *rhamnoides* × subsp. *caucasica*	[76,77]
Rapsodiia	High yield, large fruits	Ukraine, crosses with subsp. *mongolica*	[73]
Sibirsky Rumyanets	Red fruits high in carotenoids, very earrly-ly ripening	Russia	[65]
Sirola	Earrly-ly ripening	Germany, subsp. *rhamnoides* × subsp. *mongolica*	
Syurpriz Baltiki (Sjurpriz Pribaltiki)	Tolerant to wilt, medium tolerant to sea buckthorn fruit fly	Russia	[62]
Skibes siev		Latvia, from wild-growing subsp. *rhamnoides*	[54]
Sol	High yield, vigorous, round fruits, rich flavourflavor, suitable for harvesting by cutting off branches	Sweden, subsp. *mongolica* × subsp. *rhamnoides* (BHi10726)	[9]
Sudaruschka	High yield, large fruit, high carotenoid content	Russia, subsp. *mongolica*, induced chem. mutant of ‘Panteleevskaya’	[59]
Tatjana	Reddish fruits, high oil content, relatively tolerant to mycotic wilt and bud bacteriosis	Latvia, open pollination of ‘Botanicheskaya Ljubitelskaya’	[54]
Terhi	Winter hardy, high in vitamin C, tolerant to stem canker	Finland, subsp. *rhamnoides*	[76,77]
Torun	High yield, large and non-rancid fruits, suitable for harvesting by cutting off branches	Sweden, subsp. *mongolica* × subsp. *rhamnoides* (BHi727137)	
Trofimovskaya	High yield, susceptible to sea buckthorn fly	Russia, subsp. *mongolica* × subsp. *rhamnoides*	[62,72]
Tytti	Winter hardy, moderate vigor, high in vitamin C, tolerant to stem canker	Finland, subsp. *rhamnoides*	[76,77]
Vitaminnaya	Vigorous thornless bush, small fruits	Russia, subsp. *mongolica*	[59,61]
Wanhuang	High yield, late ripening yellow fruits that stay on the branches for several months	China, subsp. *mongolica* × subsp. *sinensis*	
Wanxia	High yield, late ripening orange-red fruits that stay on the branches for several months	China, subsp. *mongolica* × subsp. *sinensis*	
Wucifeng	High yield, few thorns, large fruits, long pedicels	China, offspring from subsp. *mongolica*	[70,71]
Wulanshalin	High yield, few thorns, large fruits, long pedicels	China, offspring from subsp. *mongolica*	[70,71]
Yolochka	Tolerant to wilt	Russia	[62]
Zarnitsa		Russia, subsp. *mongolica*, induced irradiation mutant	[64]
Zhemchuzhnitsa	High yield, sweet and aromatic fruits, suited for fresh consumption	Russia, subsp. *mongolica*, linebreeding of ‘Chuyskaya’	[59]
Zhongji 3	Few thorns, yellow rounded fruits	China, subsp. *mongolica* × subsp. *sinensis*	[78]
Zhongji 4	Few thorns, early ripening, yellow rounded fruits	China, subsp. *mongolica* × subsp. *sinensis*	
Zlata	High yield, thornless, large fruits, late-ripening	Russia, subsp. *mongolica*	[59]
Zolotoi Kaskad	High sugar content	Russia, subsp. *mongolica*, induced irradiation mutant	[64,65]
Zolotoi klyuchik	Tolerant to wilt, medium tolerant to sea buckthorn fruit fly	Russia	[62]

**Table 2:** Average content of phenols (mg/100 g dw) in different parts of sea buckthorn fruits, branches, and leaves of 7 cultivars derived from *H. rhamnoides* subsp. *mongolica*. Table compiled from data in Tkacz et al. [117]). SD = standard deviation, DP = degree of procyanidin polymerization, and nd = not detected.

Phenolic Group	Skin	SD	Flesh	SD	Endocarp	SD	Seeds	SD	Branches	SD	Leaves	SD
Qercetin derivatives	397.1	235.4	186.6	65.7	58.1	10.3	65.7	13.2	38.1	48.7	729.1	188.2
Isorhamnetin derivatives	308.0	208.0	114.1	62.8	72.7	18.7	112.8	24.0	3.3	3.1	173.4	36.6
Kaempferol derivatives	16.1	6.0	9.6	6.7	7.1	5.9	1.2	0.2	nd	nd	19.3	9.1
Total flavonols	721.2		310.2		137.9		179.8		41.3		921.7	
Phenolic acids	3.1	1.2	2.4	0.8	nd	nd	nd	nd	nd	nd	5.4	1.4
Flavan-3-ols	65.7	17.3	53.3	8.5	137.0	43.4	41.6	9.2	2001.3	413.6	124.7	36.8
Polymeric procyanidins	503.4	83.2	141.3	35.4	424.9	111.8	1266.8	203.2	7276.0	1574.5	974.5	533.0
DP	4.5	0.8	4.4	1.1	3.3	0.8	8.0	2.1	2.4	0.1	2.6	0.4
Total phenolics	1293.3	434.7	507.2	127.5	699.8	93.5	1488.2	241.1	9318.7	1909.5	2026.4	759.4

**Table 3:** Composition and content of phytosterols in different sea buckthorn cultivars (μg/100 mL of pulp oil). Numbers followed by the same lower-case letter constitute statistically homogeneous groups (Duncan test, *p* ≤ 0.05), and nd = not detected. Modified from Teleszko et al. [123].

Phytosterol	Aromatnaja	Avgustinka	Botaniczeskaja	Botaniczeskaja Ljubitelskaja	Luczistaja	Moskwiczanka	Podarok Sadu	Porożrachnaja
Squalene	1638 c	1205 e	2714 a	1872 b	885 g	1110 ef	1516 d	1026 f
Kampesterol	167 b	139 c	201 a	95 e	82 e	44 f	118 d	127 cd
Stigmasterol	nd	68 a	nd	nd	nd	24 b	nd	nd
β-sitosterol	6145 a	4942 b	3801 f	4022 e	2049 g	2036 g	4216 d	4409 c
Sitostanol	217 b	172 c	217 b	110 e	97 f	96 f	254 a	150 d
Δ5-avenasterol	274 b	251 c	377 a	197 d	198 d	114 e	249 c	243 c
α-amyrin	314 a	110 f	302 a	130 e	166 d	112 f	203 c	228 b
Cycloartenol	441 b	399 c	462 a	439 b	299 e	293 e	474 a	360 d
Δ7-avenasterol	156 c	152 cd	154 c	138 e	147 d	80 f	186 b	194 a
28-methylobtusifoliol	249 a	130 d	251 a	145 c	152 b	70 f	89 e	153 b
24-methylene-cycloartanol	1554 f	4048 a	2711 c	2521 d	1994 e	1454 g	3131 b	2661 c
Erythrodiol	818 a	680 b	590 c	367 f	338 g	284 h	444 d	407 e
Citrostadienol	663 a	327 d	417 c	309 e	244 g	212 h	444 b	269 f
Friedelan-3-ol	737 a	583 c	606 b	398 d	257 f	232 g	260 f	320 e
Total	13,378	13,212	12,810	10,749	6914	6168	11,592	10,553

**Table 4:** Average content of major triterpenoids (mg/100 g dw) in different parts of sea buckthorn berries, branches, and leaves of 7 cultivars derived from *H. rhamnoides* ssp. *mongolica*. Table compiled from data in Tkacz et al. [117]. SD = standard deviation.

Triterpenoids	Skin	SD	Flesh	SD	Endocarp	SD	Seeds	SD	Branches	SD	Leaves	SD
Maslinic acid	5.15	2.08	11.06	1.89	4.50	1.01	3.22	0.47	2.43	0.58	0.51	0.12
Pomolic acid	21.45	11.7	43.08	3.06	17.81	3.64	15.19	2.79	8.95	3.13	1.98	0.47
Corosolic acid	8.99	1.71	9.70	1.27	5.21	0.79	6.68	0.43	10.93	0.97	3.39	0.34
Betulinic acid	0.66	0.43	0.87	0.31	0.29	0.12	1.77	1.21	1.93	0.28	0.56	0.21
Oleanolic acid	4.73	3.59	2.65	2.93	1.41	0.97	1.85	1.22	3.70	0.33	0.63	0.08
Ursolic acid	6.45	1.53	0.46	0.09	1.31	1.35	0.39	0.06	4.28	1.00	6.24	2.70
Betulin	13.27	2.90	0.74	0.22	0.31	0.09	0.28	0.10	0.59	0.21	1.17	0.24
Total	62.26	12.76	69.61	4.97	31.3	4.55	30.21	4.15	33.75	4.34	14.72	2.41
